# A lipidome-wide association study of the lipoprotein insulin resistance index

**DOI:** 10.1186/s12944-020-01321-8

**Published:** 2020-06-25

**Authors:** Minoo Bagheri, Hemant K. Tiwari, Anarina L. Murillo, Rafet Al-Tobasei, Donna K. Arnett, Tobias Kind, Dinesh Kumar Barupal, Sili Fan, Oliver Fiehn, Jeff O’connell, May Montasser, Stella Aslibekyan, Marguerite R. Irvin

**Affiliations:** 1 Department of Epidemiology, University of Alabama at Birmingham, 1665 University Blvd, Birmingham, AL 35294 USA; 2 Department of Cardiovascular Medicine, Vanderbilt University Medical center, Nashville, TN USA; 3 Department of Biostatistics, University of Alabama at Birmingham, Birmingham, AL USA; 4 Dean’s Office, School of Public Health, University of Kentucky, Lexington, KY USA; 5 West coast metabolomics center, Davis, CA USA; 6 Department of Medicine, Division of Endocrinology, Diabetes, and Nutrition, University of Maryland, School of Medicine, Baltimore, MD USA

**Keywords:** Insulin resistance, Lipids, Lipidomics, Lipoprotein, GOLDN, Triglyceride, Diglyceride, Phospholipid

## Abstract

**Background:**

The lipoprotein insulin resistance (LPIR) score was shown to predict insulin resistance (IR) and type 2 diabetes (T2D) in healthy adults. However, the molecular basis underlying the LPIR utility for classification remains unclear.

**Objective:**

To identify small molecule lipids associated with variation in the LPIR score, a weighted index of lipoproteins measured by nuclear magnetic resonance, in the Genetics of Lipid Lowering Drugs and Diet Network (GOLDN) study (*n* = 980).

**Methods:**

Linear mixed effects models were used to test the association between the LPIR score and 413 lipid species and their principal component analysis-derived groups. Significant associations were tested for replication with homeostatic model assessment-IR (HOMA-IR), a phenotype correlated with the LPIR score (*r* = 0.48, *p* <  0.001), in the Heredity and Phenotype Intervention (HAPI) Heart Study (*n* = 590).

**Results:**

In GOLDN, 319 lipids were associated with the LPIR score (false discovery rate-adjusted *p-values* ranging from 4.59 × 10^− 161^ to 49.50 × 10^− 3^). Factors 1 (triglycerides and diglycerides/storage lipids) and 3 (mixed lipids) were positively (β = 0.025, *p* = 4.52 × 10^− 71^ and β = 0.021, *p* = 5.84 × 10^− 41^, respectively) and factor 2 (phospholipids/non-storage lipids) was inversely (β = − 0.013, *p* = 2.28 × 10^− 18^) associated with the LPIR score. These findings were replicated for HOMA-IR in the HAPI Heart Study (β = 0.10, *p* = 1.21 × 10^− 02^ for storage, β = − 0.13, *p* = 3.14 × 10^− 04^ for non-storage, and β = 0.19, *p* = 8.40 × 10^− 07^ for mixed lipids).

**Conclusions:**

Non-storage lipidomics species show a significant inverse association with the LPIR metabolic dysfunction score and present a promising focus for future therapeutic and prevention studies.

## Introduction

Dyslipidemia, one of the major determinants of cardiovascular disease (CVD) [1], is defined by elevated circulating triglycerides and decreased high-density lipoprotein (HDL) cholesterol [2]. In combination with small dense low-density lipoprotein (LDL) particles, these lipid abnormalities contribute to the insulin-resistant metabolic syndrome [2, 3], a major risk factor for type 2 diabetes (T2D). The comorbidity of insulin resistance (IR) and dyslipidemia [4] is known as diabetic dyslipidemia. Currently, the available interventions for individuals susceptible to T2D can impact IR and delay disease onset [5, 6]. However, the effectiveness of such interventions can be increased with more accurate and earlier identification of at-risk individuals, e.g. by leveraging differences in their circulating lipid patterns.

Recently, the lipoprotein insulin resistance (LPIR) score has been shown to significantly improve prediction of incident T2D in the JUPITER trial and the Women’s Health Study, even after adjustment for traditional risk factors such as smoking, physical inactivity and obesity [7–9]. The LPIR score is a novel composite metabolomic index, developed to capture the effect of IR on six lipoprotein quantities in a single algorithm [7, 8]. This score, derived from nuclear magnetic resonance (NMR) measurements, captures the accumulation of triglyceride-rich, very low-density lipoprotein particles (VLDL-P), and the consequent increase in small LDL particles (LDL-P) and reduction in large HDL particles (HDL-P) [10]. Thus, it reflects insulin-resistant dyslipoproteinemia with more precision compared to traditional lipid measures and provides stronger evidence for its association with IR than each of its individual components alone [8].

Although the LPIR score is highly variable [11], the relative contributions of genetic and environmental factors to phenotypic variation have not been comprehensively investigated. Currently evolving mass spectrometry-based lipidomics techniques, capable of detecting small lipid molecules as the lipidomic signature of lipoprotein subclasses [12], have provided insight into molecular mechanisms underlying diseases [13]. Since T2D is recognized as a global public health problem [14, 15], there is a need to develop novel prevention strategies rooted in a thorough understanding of the underlying mechanisms. The advent of high-throughput lipidomic profiling using the ultra-performance liquid chromatography coupled to (quadrupole) time-of-flight mass spectrometry (UPLC–QTOFMS) technology offers an opportunity to investigate associations between LPIR and circuating lipids, thus striving for deeper, more granular understanding of the underlying pathophysiology.

To date, there is little published research on the association between lipids and the LPIR score. As both dyslipidemia and IR are central to T2D pathogenesis, it is sensible to speculate that the differences in T2D risk can be related to the LPIR score-associated lipidomic variability. Thus, this study sought to identify a pattern of small molecule lipids associated with the LPIR score in participants of the Genetics of Lipid Lowering Drugs and Diet Network Study (GOLDN), a cohort characterized by uniquely detailed lipid assessments and a variety of –omics data. To reduce the likelihood of false positive findings inherent in the high-dimensional lipidomic analysis, a replication study was pursued in the well characterized Heredity and Phenotype Intervention (HAPI) Heart Study in which the same lipidomics data was collected.

## Methods

### Study design and population

GOLDN, the largest study of postprandial dyslipidemia that offers NMR, clinical lipid, and lipidomic measures, was initiated to assess the interaction of genetic factors with environmental interventions (intake of a high-fat meal and/or fenofibrate treatment) [16]. Briefly, the study recruited European American families with at least two siblings from two field centers (Minneapolis, MN and Salt Lake City, UT) of the Family Heart Study (FHS). Participants were excluded if they 1) had fasting triglycerides (TGs) ≥ 1500 mg/dL, 2) had a history of kidney, liver, pancreas, or gallbladder disease, recent myocardial infarction or revascularization, or nutrient malabsorption, 3) reported a current use of insulin, and 4) were pregnant or lactating. Of the 1327 participants who were initially screened, 1048 (including 546 women) met the eligibility criteria and were included in the study. A written consent form was provided for each participant and the protocol of the study was reviewed and approved by the institutional review boards at the University of Utah, University of Minnesota, and Tufts University/New England Medical Center (IRB-160331005).

### Lipoprotein phenotypes and the LPIR score

In the current study, data from participants (*n* = 980) collected at baseline after an 8-h overnight fast was used. Targeted metabolomics approach (LipoScience, Raleigh, NC) was implemented to identify NMR spectroscopy signals produced by the methyl group of lipoprotein subfractions: Large (≥ 8.8–13 nm), medium (8.2–8.8 nm) and small (7.3–8.2 nm) HDL, large (≥65 nm), medium (35–65 nm) and small VLDL (27–35 nm), and large (23–27 nm) and small (19.8–21.2 nm) LDL. LPIR is a combined weighted score of six lipoprotein subclasses or size parameters (VLDL, LDL, and HDL mean particle size; and levels of large VLDL, small LDL, and large HDL particle numbers). It was calculated for each participant using the algorithm described by Shalaurova et al. [8]. Each of six sub-scores ranges from 0 to a capped value, and the total score ranges from 0 to 100, with decreasing scores reflecting lower IR. As explained in Table 3 of the study by Shalaurova et al. [8], each sub-score reflects the six elements (VLDL, LDL, and HDL mean particle size; and levels of large VLDL, small LDL, and large HDL particle numbers). For each element, a distinct score is assigned. For example, if a participant had a VLDL size of 41.3 nm she/he received the VLDL size score of 2 corresponding to the category of “41.2–41.8”. All other five sub-scores were calculated following this procedure. The LPIR score was calculated as the sum of these six sub-scores.

### Glucose, insulin, and HOMA-IR

Laboratory assays were performed on blood samples that were collected from the study participants after an overnight fast. A hexokinase-mediated reaction on the Hitachi commercial kit (Roche Diagnostics) was used to measure fasting plasma glucose. Plasma insulin was examined using competitive RIA (Linco Research, St Charles, MO, USA). The intra-assay coefficients of variation for the above measurements were 0.984 and 0.975, respectively. HOMA-IR, used to estimate insulin resistance, was calculated as fasting plasma glucose x fasting plasma insulin/22.5 [17].

### Lipidomic phenotypes

GOLDN lipidomics data includes neutral lipids and phospholipids that were collected using UPLC–QTOFMS at the West Coast Metabolomics Center at University of California Davis, Davis, CA, US. The protocol for this measurement was described in detail elsewhere [18, 19]. Briefly, the whole process was divided into three steps: lipid extraction and separation, data acquisition and lipid identification. Methyl *tert*-butyl ether (MTBE), methanol, and water were used to extract plasma lipids. The quality control (QC) samples were method blanks and pooled human plasma (BioreclamationIVT). The separated non-polar phase was injected into a Waters Acquity UPLC CSH C18 (100 mm length × 2.1 mm id; 1.7 μm particle size) with an additional Waters Acquity VanGuard CSH C18 pre-column (5 mm × 2.1 mm id; 1.7 μm particle size) maintained at 65 °C was coupled to an Agilent 1290 Infinity UHPLC (Agilent Technologies) for ESI positive and negative modes. Mobile phase modifiers included ammonium formate and formic acid for positive mode and ammonium acetate (Sigma–Aldrich) for negative mode. The same mobile phase composition of (A) 60:40 v/v acetonitrile:water (LC-MS grade) and (B) 90:10 v/v isopropanol:acetonitrile was used for both positive and negative modes. An Agilent 6550 QTOF with a jet stream electrospray source was employed for acquiring full scan data in the mass range m/z 65–1700 in positive and negative modes with scan rate of 2 spectra/second. Instrument parameters were as follows for the ESI (+) mode – gas temperature 325 °C, gas flow 8 l/min, nebulizer 35 psig, sheath gas temperature 350 °C, sheath gas flow 11, capillary voltage 3500 V, nozzle voltage 1000 V, fragmentor voltage 120 V and skimmer 65 V. In negative ion mode, gas temperature 200 °C, gas flow 14 l/min, fragmentor 175 V, with the other parameters identical to positive ion mode. Data are collected in centroid mode at a rate of 2 scans per second. Injection volume was 1.7 μL for the positive mode and 5 μL for the negative mode. The gradient started at 15% B, ramped to 30% at 2 min, 48% at 2.5 min, 82% at 11 min, 99% at 11.5 min and kept at 99% B until 12 min before ramping down to 15% B at 12.1 min which was kept isocratic until 15 min to equilibrate the column. The total run time was 15 min and the flow rate was 0.6 ml/min. Data were acquired in nine batches and every ten samples, one quality control sample was analyzed. MS1 data were acquired for all samples, and MS/MS data were acquired for a set of pooled samples. Data were processed with the Agilent Quant 7.0 software. Lipids levels were reported as chromatographic peak heights and the data were normalized using the SERRF method (pmid 30,758,187) [20]. After normalization, the relative standard deviation of quality control samples is 4.7 and 3.4% for negative and positive mode respectively. Lipid identification was performed by converting the acquired MS/MS spectra to the mascot generic format (MGF) and then a library search using the *in-silico* MS/MS library LipidBlast.

After quality control (see supplemental material section for more details), 413 lipid compounds were included in the study.

### Replication study

The HAPI Heart Study, previously described in detail [20], was initiated in 2002 to identify the genetic and environmental determinants of responses (blood pressure, triglyceride excursion and platelet aggregation) to four short-term interventions including a cold pressor stress test, a high salt diet, a high fat challenge, and an aspirin therapy in a four-week time period. Briefly, from the 1003 individuals that were recruited from the Amish community of Lancaster County, PA into the HAPI heart study, the interventions were carried out in 868 relatively healthy Amish adults (> = 20 years of age) from large families. Participants were asked to discontinue the use of all medications, vitamins and supplements for at least 7 days prior to the first visit and during the interventions, to fast at least 12 h prior to their visit, and to restrain themselves from doing excessive physical activity on the morning of their appointment. The study protocol was approved by the Institutional Review Board of the University of Maryland, Baltimore and other participating institutions.

Fasting glucose was measured by a Beckman glucose analyzer using the glucose oxidase method, fasting insulin was examined by radioimmunoassay (Linco Research, Inc., St. Charles, MO), and HOMA-IR, was calculated as fasting plasma glucose x fasting plasma insulin/22.5. The same procedures as used in GOLDN were performed to measure small molecule lipids in the HAPI Heart study at the West Coast Metabolomics Center.

### Statistical analysis

#### Main study

Shapiro-Wilk tests were used to examine normality of the data. Quintiles of the LPIR score were compared on baseline characteristics using Chi-squared and Kruskal-Wallis tests for categorical and continuous variables, respectively. Lipid species were rank-inverse transformed to normalize the data for regression modeling. Partial correlations of LPIR score and each of its six component scores (VLDL, LDL and HDL sizes, and large VLDL, small LDL, and large HDL particle concentrations) and lipid species were estimated, adjusted for sex, age, center and body mass index (BMI). To explore associations between the LPIR score and lipid species, linear mixed models were fitted, adjusting for sex, age, study center, and BMI as fixed effects, and family structure as a random effect using the R lme4 package (*lmer* function). False discovery rate (FDR)-adjusted *p-value* <  0.05 was considered to be statistically significant in all analyses.

**Fig. 1:**
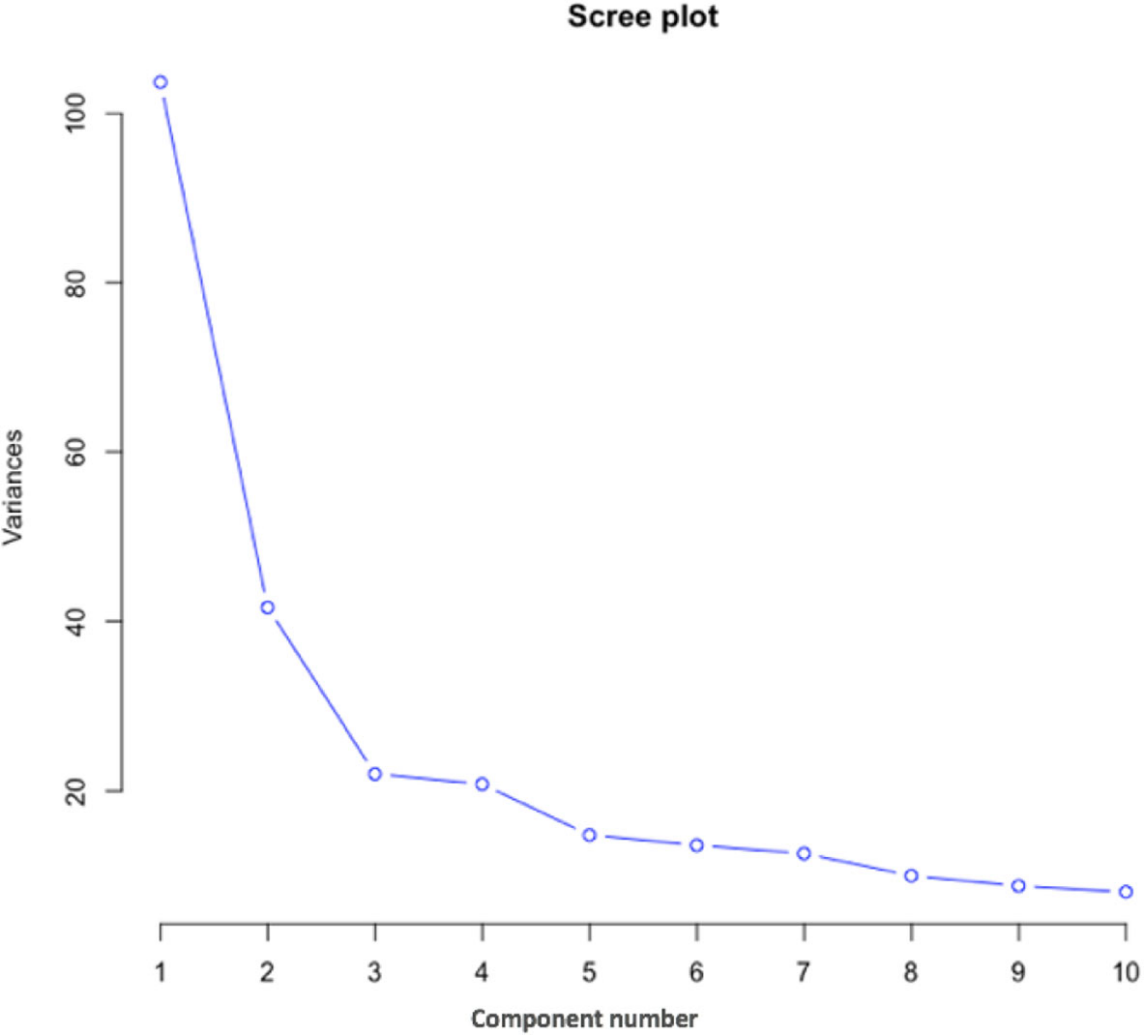
PCA scree plot indicating the explained variance of the first 10 components

Subsequently, principal component factor analysis (PFCA) was performed as a dimension reduction method to identify lipidomic patterns associated with the LPIR score. After lipid species were clustered into unrelated groups (components) using principal component analysis (PCA), three principal components were retained based on factors above the break in the scree plot (Fig. 1), to perform an exploratory factor analysis (EFA) using varimax rotation. Then the scores of each of these three factors were calculated by summing standardized variable values within each factor. Associations between the LPIR score, its component scores, and HOMA-IR with each of these three factors were tested using linear mixed models, in which sex, age, study center, and BMI were included as fixed effects and family structure was included as a random effect.

#### Replication study

A direct measure of the LPIR score was not available in HAPI Heart because NMR data were not collected in that cohort. However, because the LPIR score and HOMA-IR were strongly correlated in GOLDN (*r* = 0.48), the association of HOMA-IR with all lipid species was investigated in the replication study. Chi-squared and Kruskal-Wallis tests were used to compare categorical and continuous variables, respectively. Lipid metabolites were rank-inverse transformed since they were not normally distributed. Associations between HOMA-IR and lipid species were estimated using statistical models identical to those in the discovery stage.

All the lipids from GOLDN with loading ≥0.5 within factors 1–3 that existed in the HAPI Heart study were selected and PCA was performed on those lipids. Based on that analysis, three principal components were retained (guided by the break in the scree plot), and an exploratory factor analysis (EFA) using varimax rotation was peformed. Then the scores of each of these three factors were calculated by summing standardized variable values within each factor. Thompson’s scores were created using regression. As a result, factors 1, 2 and 3 in the HAPI Heart study consisted of polar lipids, mixed lipids, and storage lipids (triglycerides and diglycerides) respectively. The number of lipid species with factor loading exceeding 0.5 that overlapped between the GOLDN and HAPI Heart studies in the storage, non-storage and mixed lipid patterns were 40, 43, 38, respectively. The first three factors explained 41% in the metabolites in GOLDN and 55% of the variance in the HAPI Heart cohort. The association between HOMA-IR and each of the three factors were tested in HAPI Heart using models identical to the discovery analyses.

A secondary analysis was added to determine if the GOLDN LPIR associated-metabolites were also associated with HOMA-IR in GOLDN and then those lipids were compared with HOMA-IR associated lipids in the HAPI Heart study.

All data analyses were conducted in the statistical framework R 3.1.0 (www.rproject.org).

## Results

### Discovery

Table 1 shows participants’ characteristics by quintile of the LPIR score. Participants with higher LPIR scores were more likely to be male, older, and diabetic. They were also more likely to have higher BMI and waist circumference. Additionally, the level of fasting glucose, fasting insulin and HOMA-IR increased by LPIR quintile category.

**Table 1:** Characteristics^*^ of participants by quintile of the lipoprotein insulin resistance score (*n* = 980)

**Characteristics**	**Q1 (*n*** **= 198)**	**Q2 (*n*** **= 200)**	**Q3(*n*** **= 194)**	**Q4(*n*** **= 199)**	**Q5 (*n*** **= 184)**	***P*-value**
**Age at blood draw (y)**	45.0 (35.0–75.5)	46.0 (34.0–61.3)	48.0 (39.3–61.8)	50.0 (40.5–63.0)	49.5 (40.0–61.3)	0.008
**Sex**						
Male	43 (22)	82 (41)	110 (57)	106 (53)	125 (68)	< 0.001
**BMI (kg/m**^**2**^**)**	23.9 (21.6–27.4)	25.6 (23.0–28.9)	27.8 (25.5–30.9)	29.4 (27.0–32.5)	31.0 (27.8–33.8)	< 0.001
**Waist circumference (cm)**						
Male	88.0 (83.0–93.5)	93.5 (89.0–100.0)	98.0 (91.0–105.0)	103.0 (97.0–111.0)	105.0 (97.0–113.0)	< 0.001
Female	80.0 (74.0–89.5)	86.0 (79.0–94.0)	94.5 (85.8–105.3)	100.0 (91.0–110.0)	109.0 (96.5–118.0)	< 0.001
**Fasting glucose (mg/dl)**	92.0 (89.0–98.0)	94.0 (90.0–101.0)	97.5 (93.0–105.0)	101.0 (96.0–108.0)	104.0 (98.0–114.0)	< 0.001
**Fasting insulin (mU/L)**	9.0 (7.0–11.0)	10.0 (8.0–12.0)	12.0 (9.0–15.0)	15.0 (11.0–20.0)	16.0 (12.0–22.5)	< 0.001
**HOMA-IR**	2.1 (1.6–2.7)	2.5 (1.9–2.9)	2.8 (2.2–3.9)	3.6 (2.7–5.2)	4.1 (3.2–6.5)	< 0.001
**Center**						0.3
Minnesota	106 (54)	95 (48)	85 (44)	105 (53)	90 (49)	
Utah	92 (46)	105 (52)	109 (56)	94 (47)	94 (51)	
**Diabetes**						< 0.001
Yes	6 (3)	10 (5)	9 (5)	23 (12)	26 (14)	
No	192 (97)	190 (95)	184 (95)	176 (88)	158 (86)	
**Metabolic Syndrome**						0.054
Yes	76 (38)	82 (41)	64 (33)	55 (28)	63 (34)	
No	122 (62)	118 (59)	130 (67)	144 (72)	121 (66)	

* Median (IQRs) or n (%)

Abbreviations: *HOMA-IR* Homeostatic model assessment-insulin resistance

**Fig. 2:**
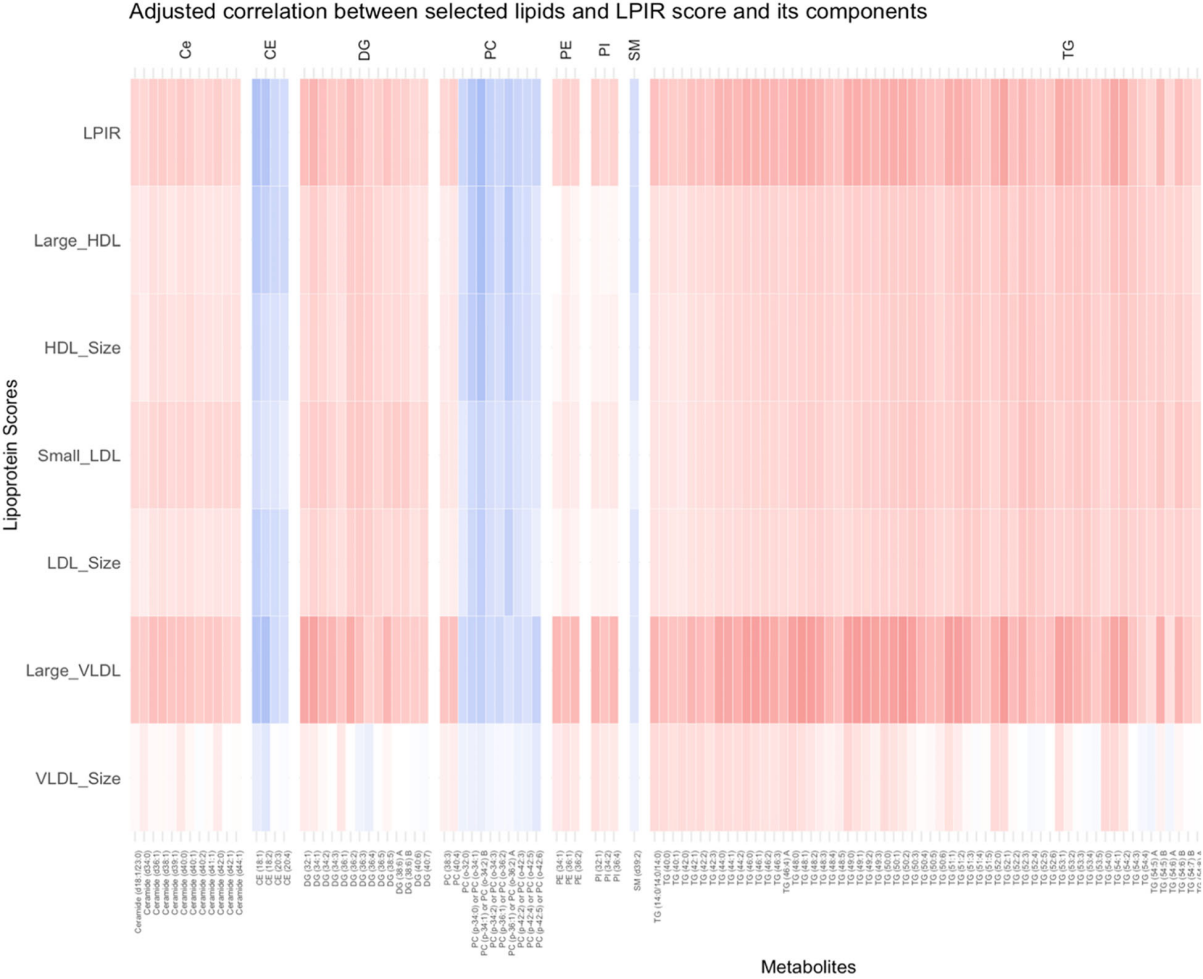
Heat map showing positive (in red), and negative (in blue) partial correlation (adjusting for sex, age, BMI and center) of the lipoprotein insulin resistance (LPIR) score and its components scores with select metabolites (those with LPIR correlation coefficient less than − 0.3 or more than 0.3 (*n* = 139)). Metabolites were characterized according to their molecular structure. Each line belongs to one metabolite. Abbreviations: *Ce = c*eramide*, CE =* cholesteryl ester*, DG =* diglycerides*, PC =* phosphatidylcholine, *PE =* phosphoethanolamine*, PI =* phosphatidylinositol*, SM =* sphingomyelin*, TG =* triglycerides

Partial correlations between the LPIR score as well as its component scores and LPIR-related lipids with *P* <  0.05 after FDR adjustment (*n* = 363 lipids)) are shown in Supplementary Figure 1. Figure 2 shows the heatmap of LPIR-correlated lipids with correlation coefficients of < − 0.3 or > 0.3 (*n* = 139). Of these LPIR-correlated lipid species, triglycerides (TGs) and diglycerides (DGs) (storage lipids), phosphatidylinositols (PIs), phosphoethanolamines (PEs), and ceramides were positively correlated while cholesteryl esters (CEs) and one single *sphingo*myelin (SM) were inversely correlated with the LPIR score. Correlations with the PCs were more heterogeneous.

Findings from the linear mixed models revealed that the LPIR score was associated (FDR-adjusted *p-value* <  0.05) with 319 lipid metabolites, controlling for age, sex, center, BMI and family relationship (Supplementary Table 1). Significantly associated lipid metabolites included 9 acylcarnitines, 7 CEs, cholesterol, 23 ceramides, 13 DGs, 25 free fatty acids, 21 lysophosphatidylcholines (LPCs), 83 phosphotidylcholines (PCs), 3 lysophosphoethanolamines (LPEs), 34 PEs, 7 prostaglandins (PGs), 5 PIs, 17 SMs, and 71 TGs (FDR-adjusted *p-values* ranging from 4.59 × 10^− 161^ to 49.50 × 10^− 3^). Supplementary Figure 2 shows the effect size and direction (derived from linear mixed models) of the significant LPIR-related metabolites grouped with respect to their molecular composition.

To identify a lipid pattern associated with LPIR and LPIR components, PCFA was performed. Lipid components with loading factors exceeding 0.5 within each factor are shown in Supplementary Table 2. Based on each factor’s constituents, their biological relevance and their loading factors, the factors were categorized as storage (factor 1), non-storage (factor 2), and mixed (factor 3) lipids. LPIR and its components were associated with storage (factor 1) and non-storage lipids (factor 2) (Table 2) with similar direction and effect size except for VLDL size and small LDL which were not associated with storage and non-storage lipids, respectively.

**Table 2:** Association^*^ of lipoprotein and insulin resistance score components with lipid species factors in the Lipid Lowering Drugs and Diet Network Study (*n* = 980)

	**Storage lipids (Factor 1)**			**Non-storage lipids (Factor 2)**			**Mixed lipids (Factor 3)**		
**Scores**	**β**	**SE**	***P*-value**^**†**^	**β**	**SE**	***P*-value**^**†**^	**β**	**SE**	***P*-value**^**†**^
LPIR	**0.025**	**0.001**	**4.52E-71**	**−0.013**	**0.001**	**2.78E-18**	**0.021**	**0.002**	**5.84E-41**
VLDL_Size	−0.004	0.004	0.356	**−0.013**	**0.004**	**0.002**	**0.046**	**0.004**	**6.06E-27**
Large_VLDL	**0.056**	**0.003**	**1.52E-65**	**−0.0161**	**0.003**	**4.11E-06**	**0.069**	**0.003**	**1.67E-96**
LDL_Size	**0.150**	**0.011**	**4.90E-43**	**−0.068**	**0.011**	**1.10E-09**	0.008	0.013	0.533
Small_LDL	**0.215**	**0.016**	**2.77E-40**	−0.028	0.017	0.096	0.033	0.018	0.096
HDL_Size	**0.066**	**0.005**	**3.62E-45**	**−0.036**	**0.005**	**6.36E-14**	0.008	0.005	0.138
Large_HDL	**0.113**	**0.007**	**2.87E-54**	**−0.079**	**0.007**	**1.05E-27**	**0.018**	**0.009**	**0.037**
HOMA-IR	**0.080**	**0.014**	**1.44E-08**	**−0.070**	**0.013**	**1.04E-07**	**0.107**	**0.015**	**6.30E-13**

*Adjusted for age, sex, BMI, center and family relationship

†*P*-values were FDR corrected to account for multiple comparisons. Bolded values indicate statistical significance (at *P*-values < 0.05)

Abbreviations: *SE* Standard error, *LPIR* Lipoprotein insulin resistance, *VLDL* Very low-density lipoprotein*, LDL* Dense low-density lipoprotein*, HDL* High-density lipoprotein*, HOMA-IR* Homeostatic model assessment-insulin resistance

### Replication

Supplementary Table 4 summarizes participants’ characteristics by quintile of HOMA-IR in the replication study. Participants with higher HOMA-IR were more likely to be female and older. Also, BMI, waist circumference and the level of fasting glucose and insulin increased by HOMA-IR quintile category.

A number of 297 lipid species overlapped between 413 and 383 compounds in the discovery and replication studies, respectively. HOMA-IR was significantly associated with 200 lipids, of which 128 overlapped with LPIR-related lipids (Supplementary Table 3). These common lipids include 5 CEs, 4 Ceramides, 6 DGs, one fatty acid, 35 PCs, 5 LPCs, 13 PEs, 12 SMs and 47 TGs. For all these lipids, the observed associations were not only significant using the FDR-corrected *p-value*, but also had the same direction of association (as evidenced by the sign of the beta) except for one ceramide, two PCs, two PEs and one SM (Ceramide (d34:2), PC (37:3), PC (p-38:2) or PC (o-38:3), PE (p-34:1) or PE (o-34:2), PE (p-38:4) or PE (o-38:5), SM (d32:2)). These lipids were directly associated with the LPIR score while they were inversely linked to HOMA-IR. Multivariate-adjusted associations of each of the three factors with HOMA-IR are shown in Table 3. Consistently with the discovery study, HOMA-IR was positively associated with storage and mixed lipids and inversely linked to non-storage lipids. Finally, secondary analysis showed that there were 105 LPIR associated metabolites that were also associated with HOMA-IR in both GOLDN and HAPI Heart. The associations were in the same direction (as evidenced by the sign of the beta) for all these common metabolites except for PC (37:3) which was inversely related to HOMA-IR in GOLDN and directly linked to HOMA-IR in the HAPI Heart study (Table 4).

**Table 3:** Association^*^ of homeostatic model assessment-insulin resistance with lipid species factors in The Heredity and Phenotype Intervention (HAPI) Heart Study (*n* = 650)

**Pattern**	**β**	**SE**	***P*-value**	**FDR-corrected*P*-value**
Non-storage lipids	− 0.13	0.04	2.10E-04	3.14E-04
Mixed lipids	0.19	0.04	2.80E-07	8.40E-07
Storage lipids	0.10	0.04	1.21E-02	1.21E-02

*Adjusted for age, sex, BMI, center and family relationship

SE: standard error

**Table 4:** Genetics of Lipid Lowering Drugs and Diet Network (GOLDN) LPIR associated lipids which are also associated with homeostatic model assessment-insulin resistance (HOMA-IR) in GOLDN compared to HOMA_IR associated lipid species in the HAPI Heart study

	**GOLDN study**			**HAPI Heart Study**		
**Metabolites**^**†**^	**β**	**SE**	**FDR-corrected*P*-value**^**‡**^	**β**	**SE**	**FDR-corrected*P*-value**^**‡**^
CE (18:1)	− 0.137	0.014	8.41E-21	−0.177	0.036	9.70E-06
CE (18:2)	−0.105	0.015	5.14E-12	−0.122	0.038	0.0043
CE (20:3)	−0.057	0.015	0.00029	−0.148	0.034	9.17E-05
CE (20:4)	−0.083	0.015	5.69E-08	−0.114	0.039	0.0086
CE (22:6)	−0.070	0.014	2.65E-06	−0.091	0.039	0.0387
Ceramide (d40:0)	0.093	0.012	4.20E-13	0.113	0.035	0.0033
Ceramide (d42:0)	0.083	0.012	1.59E-10	0.102	0.036	0.0128
DG (32:1)	0.105	0.014	3.41E-13	0.158	0.028	2.90E-07
DG (34:1)	0.115	0.013	8.72E-17	0.184	0.031	1.74E-07
DG (34:2)	0.092	0.014	1.02E-10	0.228	0.037	6.03E-08
DG (36:2)	0.070	0.014	1.76E-06	0.125	0.034	9.00E-04
DG (36:3)	0.054	0.014	0.00031	0.097	0.034	0.0123
DG (38:5)	0.061	0.014	4.79E-05	0.184	0.036	4.80E-06
GlcCer (d42:2)	−0.052	0.015	0.00078	−0.102	0.04	0.0233
LPC (18:1)	−0.051	0.014	0.00070	−0.112	0.037	0.0067
LPC (18:2)	−0.038	0.014	0.01010	−0.081	0.033	0.0291
LPC (20:1)	−0.044	0.013	0.00196	−0.149	0.035	1.00E-04
LPC (22:5)	−0.046	0.015	0.00359	−0.102	0.04	0.023
PC (34:4)	0.039	0.014	0.01240	0.088	0.036	0.0319
PC (37:3)	0.033	0.015	0.04254	−0.093	0.039	0.0345
PC (37:4)	−0.048	0.015	0.00292	−0.095	0.038	0.0281
PC (38:3)	0.061	0.015	9.58E-05	0.176	0.035	5.78E-06
PC (40:4)	0.040	0.015	0.01475	0.171	0.038	5.31E-05
PC (40:6) B	0.031	0.014	0.04002	0.1	0.038	0.0196
PC (42:5)	0.033	0.014	0.04006	0.105	0.039	0.0184
PC (42:6)	0.042	0.015	0.00878	0.135	0.037	0.001
PC (o-32:0)	−0.040	0.015	0.01473	−0.181	0.035	2.45E-06
PC (p-32:0) or PC (o-32:1)	−0.058	0.015	0.00031	−0.221	0.036	4.60E-08
PC (p-34:0) or PC (o-34:1)	−0.095	0.014	9.43E-11	−0.244	0.034	3.85E-10
PC (p-34:1) or PC (o-34:2) B	−0.141	0.013	4.08E-24	−0.302	0.032	6.01E-17
PC (p-34:2) or PC (o-34:3)	−0.090	0.015	4.09E-09	−0.252	0.035	2.28E-10
PC (p-36:1) or PC (o-36:2) A	−0.092	0.013	0.02546	−0.19	0.039	1.31E-05
PC (p-36:2) or PC (o-36:3)	−0.044	0.015	0.00736	−0.177	0.036	1.13E-05
PC (p-36:4) or PC (o-36:5)	−0.054	0.014	0.00044	−0.172	0.038	4.84E-05
PC (p-38:4) or PC (o-38:5) A	−0.058	0.015	0.00032	−0.136	0.038	0.0013
PC (p-38:4) or PC (o-38:5) B	−0.068	0.014	6.66E-06	−0.218	0.036	8.86E-08
PC (p-40:1) or PC (o-40:2)	−0.035	0.015	0.03400	−0.115	0.039	0.0082
PC (p-40:3) or PC (o-40:4)	−0.042	0.015	0.00917	−0.157	0.037	1.00E-04
PC (p-40:4) or PC (o-40:5)	−0.055	0.015	0.00049	−0.198	0.037	1.54E-06
PC (p-40:6) or PC (o-40:7) B	−0.031	0.014	0.04752	−0.186	0.038	1.28E-05
PC (p-42:2) or PC (o-42:3)	−0.069	0.014	4.93E-06	−0.173	0.04	7.84E-05
PC (p-42:3) or PC (o-42:4)	−0.053	0.015	0.00089	−0.111	0.04	0.0123
PC (p-42:4) or PC (o-42:5)	−0.060	0.015	0.00012	−0.201	0.037	1.12E-06
PC (p-42:5) or PC (o-42:6) A	−0.045	0.015	1.07E-06	−0.168	0.04	1.00E-04
PC (p-44:4) or PC (o-44:5)	−0.053	0.015	0.00063	−0.187	0.036	3.09E-06
PC (p-44:5) or PC (o-44:6)	−0.058	0.015	0.00039	−0.121	0.036	0.0028
PE (34:1)	0.080	0.013	1.07E-08	0.116	0.033	0.0017
PE (34:2)	0.073	0.014	2.10E-06	0.129	0.032	3.00E-04
PE (36:1)	0.114	0.014	2.66E-15	0.127	0.027	1.76E-05
PE (36:2)	0.104	0.014	1.40E-13	0.135	0.029	2.09E-05
PE (36:3)	0.069	0.014	4.25E-06	0.06	0.026	0.04
PE (36:4)	0.034	0.013	0.02289	0.092	0.035	0.0196
PE (38:4) B	0.052	0.012	6.71E-05	0.084	0.038	0.0483
PE (38:6)	0.040	0.013	0.00539	0.077	0.034	0.0476
PE (p-40:5) or PE (o-40:6)	−0.037	0.014	0.01636	−0.1	0.042	0.0348
SM (d38:2)	−0.058	0.014	0.00015	−0.171	0.033	2.43E-06
SM (d39:2)	−0.074	0.014	3.75E-07	−0.18	0.034	1.54E-06
SM (d40:2) B	−0.056	0.015	0.00048	−0.157	0.037	1.00E-04
SM (d40:3)	−0.043	0.014	0.00566	−0.197	0.036	1.27E-06
SM (d42:0)	0.040	0.014	0.00740	0.098	0.038	0.023
SM (d42:2) B	−0.038	0.014	0.01437	−0.167	0.037	4.84E-05
SM (d42:3)	−0.054	0.013	0.00010	−0.224	0.036	2.54E-08
TG (40:1)	0.124	0.014	6.21E-17	0.057	0.024	0.0336
TG (42:0)	0.117	0.015	4.86E-14	0.08	0.024	0.0024
TG (42:1)	0.118	0.015	1.01E-14	0.082	0.024	0.0017
TG (44:1)	0.132	0.014	6.00E-19	0.101	0.024	1.00E-04
TG (44:2)	0.133	0.014	6.13E-19	0.116	0.024	1.50E-05
TG (46:0)	0.142	0.014	4.69E-23	0.092	0.024	6.00E-04
TG (46:1)	0.135	0.014	1.33E-20	0.131	0.024	8.49E-07
TG (46:2)	0.132	0.014	5.69E-19	0.136	0.025	7.46E-07
TG (48:0)	0.138	0.014	3.64E-22	0.147	0.028	1.54E-06
TG (49:0)	0.139	0.014	3.00E-22	0.076	0.024	0.005
TG (49:1)	0.133	0.014	2.06E-20	0.115	0.025	2.17E-05
TG (49:3)	0.107	0.014	6.96E-13	0.136	0.03	3.87E-05
TG (50:1)	0.132	0.014	8.68E-21	0.183	0.027	1.77E-09
TG (50:2)	0.121	0.013	2.59E-18	0.189	0.029	1.31E-08
TG (51:1)	0.129	0.014	1.29E-19	0.11	0.024	3.57E-05
TG (51:2)	0.112	0.014	3.28E-15	0.133	0.029	3.79E-05
TG (51:3)	0.091	0.014	5.44E-10	0.125	0.034	9.00E-04
TG (51:4)	0.076	0.015	1.09E-06	0.109	0.034	0.0041
TG (51:5)	0.071	0.015	5.72E-06	0.147	0.036	3.00E-04
TG (52:1)	0.143	0.013	2.38E-25	0.15	0.024	3.79E-08
TG (52:2)	0.062	0.014	4.71E-05	0.163	0.034	1.28E-05
TG (52:3)	0.085	0.014	3.51E-09	0.142	0.035	3.00E-04
TG (52:4)	0.072	0.014	1.78E-06	0.199	0.035	2.51E-07
TG (52:6)	0.065	0.015	2.81E-05	0.217	0.037	1.94E-07
TG (53:1)	0.138	0.014	1.08E-22	0.098	0.023	2.00E-04
TG (53:3)	0.083	0.014	1.74E-08	0.11	0.033	0.0032
TG (53:4)	0.073	0.014	1.79E-06	0.125	0.036	0.002
TG (53:5)	0.055	0.015	0.00048	0.118	0.035	0.0024
TG (54:0)	0.111	0.015	2.43E-13	0.084	0.034	0.0309
TG (54:1)	0.142	0.014	1.47E-23	0.123	0.024	2.45E-06
TG (54:3)	0.060	0.014	7.07E-05	0.095	0.035	0.0177
TG (55:3)	0.083	0.014	2.77E-08	0.081	0.03	0.0168
TG (56:2)	0.125	0.014	1.82E-18	0.141	0.029	8.44E-06
TG (56:4)	0.059	0.015	0.00019	0.1	0.035	0.0119
TG (56:7) B	0.063	0.014	1.78E-05	0.127	0.036	0.0013
TG (56:8) B	0.061	0.014	4.45E-05	0.115	0.036	0.0039
TG (57:2)	0.127	0.014	1.39E-18	0.091	0.025	0.0013
TG (58:1)	0.109	0.014	1.12E-13	0.167	0.031	1.47E-06
TG (58:2)	0.112	0.014	3.38E-14	0.163	0.037	5.74E-05
TG (58:3)	0.097	0.014	8.15E-11	0.149	0.042	0.0015
TG (58:5)	0.073	0.015	3.67E-06	0.128	0.035	0.001
TG (58:6)	0.071	0.014	2.42E-06	0.14	0.034	2.00E-04
TG (60:2)	0.092	0.014	4.06E-10	0.128	0.036	0.0014

*Adjusted for age, sex, BMI, center and family relationship

† Metabolite values were rank-inverse transferred

‡ *P*-values were FDR corrected to account for multiple comparisons. Results are shown for significant metabolites (FDR-adjusted *P* < 0.05). study

Abbreviations: *SE* Standard Error*, CE* Cholesteryl ester*, DG* Diglycerides*, LPC* Lysophosphatidylcholine*, PC* Phosphatidylcholine, *PE* Phosphoethanolamine*, SM* Sphingomyelin*, TG* Triglycerides

## Discussion

In the current research, which was the first comprehensive lipidomic study of the LPIR score, statistically significant associations with several classes of lipids were found. Specifically, TGs, DGs, PIs, PEs and ceramides were positively, and CEs and one SM were inversely and strongly related to this measure of metabolic dysfunction. Furthermore, metabolites’ patterns characterized by PCFA distinguished storage and non-storage lipids that were directly and inversely associated with the score, respectively. These patterns provide the first evidence of molecular distinctions between various levels of LPIR-assessed metabolic dysfunction. These findings were validated using a related phenotype (HOMA-IR) in an independent population characterized using the same lipidomics approach, reducing the chance of false positive findings.

Findings of this study are concordant with several previous reports of lipid associations with IR, prediabetes and T2D [21–23]. For example, TGs have been previously proposed as the early markers of T2D [23]. Concordantly, a robust direct association between TGs with shorter chain fatty acids and the LPIR score was observed. Similarly, a previous metabolomic study reported that TGs containing shorter chain fatty acids were elevated in pre-diabetes and T2D, while TGs with longer chain fatty acids were associated with a decreased risk of these metabolic disorders [24]. On the other hand, when not esterified, fatty acids act differently. Importantly, short chain free fatty acids were reported to be depleted in diabetic patients while medium and long chain free fatty acids were higher in patients with impaired fasting glucose (IFG) and T2D compared to controls [24, 25]. The current study showed that saturated and longer chain free fatty acids were directly associated with the LPIR score. Also, in compounds with the same carbon number, fatty acids with more double bonds showed lower effect sizes in their relationships with the LPIR score in comparison with more saturated fatty acids. Impaired insulin function can be stimulated by fatty acids through mechanisms including inflammation, oxidative stress, mitochondrial dysfunction and the accumulation of lipid derivatives [26].

The assessment of the fatty acid composition of cholesterol esters provides important information about a potential role in health and disease. In this study, cholesterol esters containing fatty acids with carbon number greater or equal to 18 were inversely associated with the LPIR score. Consistently, other research has shown that in groups with impaired glucose tolerance or diabetes compared to those with normal glucose tolerance, the proportion of palmitic acid (16:0) and palmitoleic acid (16:1) in serum cholesterol esters was higher while the proportion of linoleic acid (18:2) was lower [27]. Similarly, as indicated in Supplementary Table 5, CE (16:1) was directly correlated with lipid measurements (HDL, LDL, total cholesterol and TG) and insulin resistance and cholesterol esters having fatty acids with greater carbon numbers were inversely correlated with TG and insulin resistance. In this study, individuals with higher LPIR scores had elevated levels of free cholesterol and reduced levels of all forms of cholesteryl esters. Cholesteryl ester transfer protein (CETP), a protein involved in replacing lipids between lipoproteins, improves insulin sensitivity in obesity through increased cholesterol delivery to liver and activation of bile acid-sensitive pathways [28]. This could explain the inverse relationship, observed in the current study, especially for CEs 18:1, 18:2, 20:3 and 20:4.

Other studies have also reported phospholipids as markers of either diabetes or the complications associated with this metabolic dysfunction [21, 22]. Findings of this study have also revealed that in the PCs group, there were a number of species associated with a higher LPIR score, while other compounds called PCs isobars or PC-O lipid species or ether PCs (e.g. PC (p-36:4) or PC (o-36:5)) were associated with a decreased risk of metabolic dysfunction. Consistent with these findings, there is other research suggesting that PC-Os were lower in individuals with hypertension compared to normotensive controls [29]. It is currently unclear whether PC-Os have an ameliorating effect on IR and subsequent metabolic complications or whether reduced risk factor accompanied by a healthy status would contribute to diminished ether PCs. However, their association with the LPIR score was discrepant from the other PCs. This observation might be due to their structural variation, especially in their fatty acid side chain (based on carbon number and double bond) between these two sub-groups of lipid species. Previous research highlighted the importance of fatty acid composition within phospholipid molecules, including PCs and PEs in determining insulin responsiveness [30]. More specifically, phospholipids containing longer chain and highly unsaturated fatty acids were related to reduced cardiometabolic risks.

Regarding LPCs, which are produced when phospholipids such as PCs or PEs are hydrolyzed by phospholipase A_2_ (PLA_2_) [31], results from published studies vary. In harmony with the findings of this study, lower levels of some LPC species including LPC (18:2) were associated with a higher risk of metabolic dysfunction [32, 33]. However, there were some species like LPC (16:1) that were directly related to metabolic dysfunction [33]. While observed differences in the relationship of LPCs with metabolic disease could be due to the fatty acid side chain, PLA_2_ isoforms could also play a role. For example, to protect from adipose tissue inflammation during obesity, hyperlipidemic LDL is hydrolyzed by PLA_2_-V to release unsaturated fatty acids which aid saturated adipocytes-released fatty acids to initiate the polarization of macrophages [31].

Ceramides, compounds composing of a sphingosine and a fatty acid, were directly associated with higher LPIR scores. Bergman et al. pointed to C18:0, C20:0, and C24:1 ceramides that were increased in T2D, and C16:0 ceramide that was elevated in patients with IR [34]. Impaired insulin function can be partly attributed to the increased levels of intracellular lipids such as DGs and ceramides [26]. The same study also suggested that while SM C18:0 positively correlated with insulin resistance, other SM species (C14:0, C22:3, and C24:4) are positively related to insulin secretion [34]. This means that with respect to SMs, which are ceramides with a PC within the molecule, not all species showed a similar trend in metabolic dysregulation. Also, patients with IFG and T2D were reported to have higher levels of SMs compared to healthy people [26]. Consistently with prior literature, findings from this study demonstrated that some SMs were elevated and some were diminished in participants with higher measures of metabolic dysfunction; among 17 LPIR score- related SMs, SM (d39:2) exhibited the strongest and inverse association. Reduced SM synthesis underlies accumulation of reactive oxygen species, which can lead to pancreatic β-cell dysfunction and cause reduced insulin secretion [35].

Given that many correlated metabolites can reside in overlapping pathways, there is value in investigating patterns captured by particular clusters of metabolites. Of the three factors that were assessed in the current study, the relevance of storage lipids and its components to the score and its role in metabolic disease etiology is of particular interest. Specifically, levels of storage lipids, composed of TGs and DGs (storage lipids), increased with the LPIR score. Similar significant patterns of this association were found for all components of the LPIR score, except for VLDL particle size. A consistent finding, with respect to different types of lipoproteins, has been reported during IR status and diabetes [36, 37]. However, VLDL particle size has also been increased in this situation [36]. The discrepancy regarding this particular lipoprotein metric might be due to the general good health status of GOLDN participants; 70% of the study population had optimal levels of IR (HOMA-IR ≤ 3.8 [38]), and 92.5% of them were non-diabetic. Plasma lipoprotein fractions may contribute to the transition to IR status. Elevated large VLDL is the primary abnormality involved in increasing small-dense LDL production [36, 37], and results of this study suggest both large VLDL and small LDL are strongly associated with storage lipids and thus could be significant in developing IR [39].

In contrast to factor 1, factor 2, composed of non-storage lipids and polar lipids, was inversely associated with LPIR and all its subclass scores, except for small LDL. A non-significant association of non-storage lipid pattern with the small LDL score might indicate that it is TGs but not phospholipids component of LDL that play an important role in progression of IR and T2D in healthy individuals. The observed relationship between the LPIR score and lipid pattern could be described in terms of the lipid constituents of this pattern. The storage lipid pattern was mostly composed of TGs which are positively related to increased metabolic dysfunction including diabetes [40]. Further, the majority of lipids with high loading factor within non-storage lipid pattern included SMs and PCs which are protective effects against metabolic dysfunction [35]. The observation that the mixed lipid pattern (factor 3) was mostly composed of TGs and DGs likely explains the direct association between the LPIR score and the third lipid pattern.

### Study limitation

The findings should be considered in the context of several limitations. First, the cross-sectional study cannot establish a temporal or causal relationship between lipid species and the LPIR score. Secondly, participants of the GOLDN study were largely metabolically healthy Americans of European descent, which might limit the generalizability of the findings to other ethnic groups and clinical populations. Finally, even though the findings were reported after controlling for potential confounders including family relationship, residual confounding may not be excluded such as for age and/or gender.

Also, while we were able to replicate many of the lipids discovered in GOLDN for LPIR score using HOMA-IR as the outcome in the HAPI Heart study, HOMA-IR was not a perfect proxy. However, secondary analysis of LPIR associated lipids showed the majority were also associated with HOMA-IR in GOLDN lending support to using that phenotype in the external replication effort. Furthermore, these two cohorts were different based on gender. This discrepancy is likely explained by inherent differences in the GOLDN and HAPI Heart populations, especially since the latter is an isolated Amish population. However, Using the HAPI Heart Study was the best opportunity for replication given the Amish cohort is of comparable size and race and had the same lipidomic assays conducted at the same lab (West Coast Metabolomics Center). To address this difference in the analysis, sex was adjusted for in all analytical models and many of the results did replicate.

### Study strength

In spite of these limitations, the study possesses some major strengths. First, GOLDN and HAPI Heart were large well characterized studies of Caucasian adults that had available clinical metabolic data and lipidomic data from the same lab enabling external validation of the findings. Secondly, a lipidome-wide approach was employed to comprehensively characterize all associations, compared to previous smaller candidate lipid studies. This study was the first to describe molecular correlates of higher LPIR scores prior to dysglycemia onset, and these findings provide the first evidence of potential lipid targets for interventions [41, 42].

## Conclusion

a strong positive association was found between storage lipids including TGs and DGs and a strong inverse association was observed between non-storage lipids with the metabolic dysfunction score in a healthy population.

Clinical relevance: Additional research should evaluate whether storage and non-storage lipid patterns, especially among those with optimal clinical profiles but a higher LPIR score, could be useful to inform metabolic disease prevention.

Take home message: A pattern of higher storage lipids (e.g. TGs) and lower non-storage lipids (e.g. PCs) is associated with higher LPIR score and insulin resistance in Caucasian adults. More studies are needed to determine if these lipid patterns could offer early targets for prevention of metabolic disease.

## Supplementary information

**Additional file 1. Supplementary Table 1.** Linear mixed models for the association of the lipoprotein insulin resistance score with plasma lipids in the *Lipid Lowering Drugs and Diet Network* study. **Supplementary Table 2.** Factors identified from exploratory factor analysis following principal components analysis (PCA) in the *Lipid Lowering Drugs and Diet Network* study of lipidomics. Only metabolites with a factor loading ≥ 0.5 were reported as composing a given factor. **Supplementary Table 3.** Results of associations between homeostatic model assessment-insulin resistance and plasma lipids in the HAPI Heart study. **Supplementary Table 4.** Characteristics of participants by quintile of the homeostatic model assessment-insulin resistance (*n* = 590). **Supplementary Table 5.** Partial correlation between cholesterol esters and lipids and glycemic measurements. **Supplementary Figure 1. **Heat map showing positive (in red), and negative (in purple) partial correlations (adjusting for sex, age, BMI and center) of the lipoprotein insulin resistance (LPIR) score and its component scores with LPIR-correlated metabolites (*n* = 363); metabolites were characterized according to their molecular structure. Each line belongs to one metabolite. **Supplementary Figure 2.** Bar plot showing positive (in blue) and negative (in red) effect size derived from linear mixed models of the significant lipoprotein insulin resistance (LPIR)-related metabolites (*n* = 319) characterized with respect to metabolite composition. Each line belongs to one metabolite. To have a better visualization groups with one metabolite (cholesterol and lactosylceramide (d18:1/24:1(15Z))) were not included in the figure.

**Additional file 2: Supplementary Table 6.** Lipidomcs data for UPLC-QTOF/MS.

## Data Availability

Data for UPLC-QTOF/MS are attached as Supplementary Table 6.

## Abbreviations

CE: Cholesteryl esters
CETP: Cholesteryl ester transfer protein
DG: Diglyceride
EFA: Exploratory factor analysis
FDR: False discovery rate
FHS: Family heart study
GOLDN: Genetics of lipid lowering drugs and diet network
HAPI: Heredity and phenotype intervention
HDL-P: HDL particles
HOMA-IR: Homeostatic model assessment-insulin resistance
IFG: Impaired fasting glucose
IR: Insulin resistance
LDL-P: LDL particles
LPIR: Lipoprotein insulin resistance
MGF: Mascot generic format
MTBE: Methyl *tert*-butyl ether
PCA: Principal component analysis
PCFA: Principal component factor analysis
QC: Quality control
T2D: Type 2 diabetes
UPLC–QTOFMS: Ultra-performance liquid chromatography coupled to quadrupole time-of-flight mass spectrometry
VLDL-P: VLDL particles
