# Supplementary material for: A lipidome-wide association study of the lipoprotein insulin resistance index
Source: Lipids Health Dis. 2020 Jun 25;19:153. doi: 10.1186/s12944-020-01321-8 (PMC7318473; doi:10.1186/s12944-020-01321-8)
Supplement: Supplementary file 1 — Additional file 1. Supplementary Table 1. Linear mixed models for the association of the lipoprotein insulin resistance score with plasma lipids in the Lipid Lowering Drugs and Diet Network study. Supplementary Table 2. Factors identified from exploratory factor analysis following principal components analysis (PCA) in the Lipid Lowering Drugs and Diet Network study of lipidomics. Only metabolites with a factor loading ≥ 0.5 were reported as composing a given factor. Supplementary Table 3. Results of associations between homeostatic model assessment-insulin resistance and plasma lipids in the HAPI Heart study. Supplementary Table 4. Characteristics of participants by quintile of the homeostatic model assessment-insulin resistance (n = 590). Supplementary Table 5. Partial correlation between cholesterol esters and lipids and glycemic measurements. Supplementary Figure 1. Heat map showing positive (in red), and negative (in purple) partial correlations (adjusting for sex, age, BMI and center) of the lipoprotein insulin resistance (LPIR) score and its component scores with LPIR-correlated metabolites (n = 363); metabolites were characterized according to their molecular structure. Each line belongs to one metabolite. Supplementary Figure 2. Bar plot showing positive (in blue) and negative (in red) effect size derived from linear mixed models of the significant lipoprotein insulin resistance (LPIR)-related metabolites (n = 319) characterized with respect to metabolite composition. Each line belongs to one metabolite. To have a better visualization groups with one metabolite (cholesterol and lactosylceramide (d18:1/24:1(15Z))) were not included in the figure. [file 12944_2020_1321_MOESM1_ESM.docx]

**A lipidome-wide association study of the lipoprotein insulin resistance index**

Authors: Minoo Bagheri^1,2^, Hemant K. Tiwari^3^, Anarina Murillo^4^, Rafet Al-Tobasei^5^, Donna K. Arnett^6^, Tobias Kind^7^, Dinesh Kumar Barupal^8^, Sili Fan^9^, Oliver Fiehn^10^, Jeff O'connell^11^, May Montasser^12^, Stella Aslibekyan^13^, Marguerite Ryan Irvin^14^

1. Department of Epidemiology, University of Alabama at Birmingham, Birmingham, AL, US, bagherim@uab.edu
2. Department of Cardiovascular Medicine, Vanderbilt University Medical center, minoo.bagheri@vumc.org
3. Department of Biostatistics, University of Alabama at Birmingham, Birmingham, AL, US, [htiwari@uab.edu](mailto:htiwari@uab.edu)
4. Department of Biostatistics, University of Alabama at Birmingham, Birmingham, AL, US, amurillo@uab.edu
5. Department of Biostatistics, University of Alabama at Birmingham, Birmingham, AL, US, rtobasei@uab.edu
6. Department of Epidemiology, University of Kentucky, Lexington, KY, US, donna.arnett@uky.edu
7. West coast metabolomics center, Davis, CA, US, tkind@ucdavis.edu
8. West coast metabolomics center, Davis, CA, US, dinkumar@ucdavis.edu
9. West coast metabolomics center, Davis, CA, US, slfan@ucdavis.edu
10. West coast metabolomics center, Davis, CA, US, [ofiehn@ucdavis.edu](mailto:ofiehn@ucdavis.edu)
11. Department of Medicine, University of Maryland, Collage park, Maryland, US, joconnel@som.umaryland.edu
12. Department of Medicine, University of Maryland, Collage park, Maryland, US, mmontass@som.umaryland.edu
13. Department of Epidemiology, University of Alabama at Birmingham, Birmingham, AL, US, [saslibek@uab.edu](mailto:saslibek@uab.edu)
14. Department of Epidemiology, University of Alabama at Birmingham, Birmingham, AL, US, [irvinr@uab.edu](mailto:irvinr@uab.edu)

Methods:

Quality control analysis for lipidomics data:

The quality control for lipidomic profiles are multilayered. Instruments are mass calibrated before every batch run. Fourteen deuterated internal standards each representing different lipid classes are added to each single sample. These internal standards are utilized to follow deviations and assess reproducibility, repeatability and other analytical figures of merit. Some of the internal standards are used for retention time corrections, others for quantification only. Additionally, every 11th sample is a pooled quality control (QC) sample to follow deviations across large batches. Every batch also contains several NIST SRM 1950 plasma QC samples (standard reference material SRM 1950; Metabolites in Frozen Human Plasma; NIST, Gaithersburg, MD) which allow for additional external validation of the pooled plasma QC samples. Additional software procedures that include normalization protocols are then used to assess and correct for observed technical issues. (For further details see: Cajka et al: Anal. Chem. 2017, 89, 12360-12368; Validating Quantitative Untargeted Lipidomics Across Nine Liquid Chromatography-High-Resolution Mass Spectrometry Platforms).

| Supplementary Table 1-Linear mixed models for the association^*^ of the lipoprotein insulin resistance score with plasma lipids in the Lipid Lowering Drugs and Diet Network study | | | |
| --- | --- | --- | --- |
| **Metabolites**^†^ | **β** | **SE** | **FDR-corrected P-value**^‡^ |
| Acylcarnitine (C10:1) | -0.008 | 0.002 | 6.38E-06 |
| Acylcarnitine (C14:2) | -0.010 | 0.002 | 2.50E-09 |
| Acylcarnitine (C18:3) | -0.005 | 0.002 | 0.0022 |
| Acylcarnitine (C8:0) | -0.006 | 0.002 | 0.0012 |
| Acylcarnitine C10:0 | -0.005 | 0.002 | 0.0038 |
| Acylcarnitine C12:0 | -0.005 | 0.002 | 0.0027 |
| Acylcarnitine C16:0 | 0.004 | 0.002 | 0.0416 |
| Acylcarnitine C18:1 | -0.006 | 0.002 | 0.0007 |
| Acylcarnitine C18:2 | -0.010 | 0.002 | 7.93E-10 |
| CE (18:1) | -0.026 | 0.001 | 1.77E-77 |
| CE (18:2) | -0.026 | 0.001 | 2.28E-72 |
| CE (18:3) | -0.012 | 0.002 | 1.47E-12 |
| CE (20:3) | -0.017 | 0.002 | 7.75E-27 |
| CE (20:4) | -0.017 | 0.002 | 3.47E-26 |
| CE (20:5) | -0.007 | 0.002 | 1.19E-05 |
| CE (22:6) | -0.015 | 0.002 | 7.44E-23 |
| Ceramide (d18:1/23:0) | 0.015 | 0.001 | 4.14E-22 |
| Ceramide (d32:1) | 0.011 | 0.002 | 1.03E-30 |
| Ceramide (d33:1) | 0.008 | 0.002 | 2.69E-14 |
| Ceramide (d34:0) | 0.014 | 0.002 | 5.82E-11 |
| Ceramide (d34:1) | 0.011 | 0.001 | 0.0039 |
| Ceramide (d34:2) | 0.001 | 0.001 | 6.73E-68 |
| Ceramide (d36:1) | 0.016 | 0.001 | 9.52E-90 |
| Ceramide (d38:1) | 0.017 | 0.001 | 1.37E-51 |
| Ceramide (d39:1) | 0.016 | 0.001 | 3.50E-41 |
| Ceramide (d40:0) | 0.017 | 0.001 | 1.72E-34 |
| Ceramide (d40:1) | 0.016 | 0.001 | 1.93E-71 |
| Ceramide (d40:2) | 0.013 | 0.002 | 1.19E-41 |
| Ceramide (d41:1) | 0.014 | 0.001 | 4.97E-22 |
| Ceramide (d42:0) | 0.015 | 0.001 | 1.56E-31 |
| Ceramide (d42:1) | 0.011 | 0.001 | 8.10E-06 |
| Ceramide (d42:2) A | 0.011 | 0.001 | 1.75E-10 |
| Ceramide (d42:2) B | 0.010 | 0.002 | 2.08E-46 |
| Ceramide (d43:1) | 0.010 | 0.002 | 2.60E-27 |
| Ceramide (d44:1) | 0.011 | 0.001 | 2.96E-25 |
| Cholesterol | -0.005 | 0.002 | 2.74E-19 |
| DG (32:1) | 0.024 | 0.001 | 5.14E-17 |
| DG (34:1) | 0.026 | 0.001 | 3.18E-12 |
| DG (34:2) | 0.021 | 0.001 | 0.0160 |
| DG (34:3) | 0.020 | 0.001 | 0.0039 |
| DG (36:1) | 0.019 | 0.002 | 3.31E-08 |
| DG (36:2) | 0.024 | 0.001 | 1.02E-13 |
| DG (36:3) | 0.020 | 0.001 | 0.0219 |
| DG (36:4) | 0.015 | 0.002 | 0.0012 |
| DG (38:2) | 0.003 | 0.002 | 0.0358 |
| DG (38:3) | 0.011 | 0.002 | 0.0080 |
| DG (38:5) | 0.021 | 0.001 | 0.0058 |
| DG (38:6) A | 0.017 | 0.002 | 9.59E-05 |
| DG (40:6) | 0.015 | 0.002 | 9.42E-07 |
| FA (12:0) | 0.003 | 0.002 | 0.0256 |
| FA (14:1) | -0.001 | 0.002 | 0.0253 |
| FA (15:0) A | 0.001 | 0.002 | 8.40E-05 |
| FA (15:0) B | 0.002 | 0.002 | 0.0003 |
| FA (15:1) | 0.000 | 0.002 | 0.0002 |
| FA (16:0) | 0.003 | 0.002 | 0.0141 |
| FA (16:1) | -0.001 | 0.002 | 2.53E-19 |
| FA (17:1) (heptadecenoic acid) | 0.001 | 0.002 | 6.88E-24 |
| FA (18:0) | 0.004 | 0.002 | 1.20E-31 |
| FA (18:1) | -0.001 | 0.002 | 0.0002 |
| FA (18:2) | -0.003 | 0.002 | 1.37E-16 |
| FA (18:3) | -0.002 | 0.002 | 2.26E-24 |
| FA (19:0) | 0.002 | 0.002 | 3.39E-22 |
| FA (20:0) (arachidic acid) | 0.003 | 0.002 | 3.61E-09 |
| FA (20:1) (eicosenoic acid) | -0.002 | 0.002 | 1.73E-08 |
| FA (20:2) (eicosadienoic acid) | -0.002 | 0.002 | 4.90E-28 |
| FA (20:4) | 0.001 | 0.002 | 4.84E-07 |
| FA (20:5) | -0.002 | 0.002 | 9.84E-15 |
| FA (20:6) | -0.002 | 0.002 | 4.05E-12 |
| FA (22:1) (erucic acid) | 0.001 | 0.002 | 2.69E-08 |
| FA (22:6) | -0.002 | 0.002 | 0.0014 |
| FA (24:0) (lignoceric acid) | 0.005 | 0.002 | 1.12E-10 |
| FA (24:1) (nervonic acid) | 0.003 | 0.002 | 0.0220 |
| FA (26:0) (cerotic acid) | 0.008 | 0.002 | 1.17E-09 |
| FA (28:0) (montanic acid) | 0.005 | 0.002 | 0.0292 |
| GlcCer (d42:1) | -0.005 | 0.002 | 9.86E-08 |
| GlcCer (d42:2) | -0.009 | 0.002 | 2.28E-21 |
| GlcCer(d14:1(4E)/20:0(2OH)) | 0.001 | 0.001 | 0.0086 |
| Lactosylceramide (d18:1/24:1(15Z)) | -0.012 | 0.002 | 6.37E-05 |
| LPC (14:0) | 0.014 | 0.002 | 0.0240 |
| LPC (15:0) | 0.004 | 0.002 | 3.30E-36 |
| LPC (16:1) | 0.009 | 0.002 | 3.64E-06 |
| LPC (17:1) | 0.002 | 0.002 | 0.0385 |
| LPC (18:0) | 0.002 | 0.002 | 6.69E-05 |
| LPC (18:0) A | 0.001 | 0.002 | 1.72E-05 |
| LPC (18:1) | -0.003 | 0.002 | 4.20E-12 |
| LPC (18:2) | -0.006 | 0.002 | 1.62E-11 |
| LPC (18:3) | 0.005 | 0.002 | 0.0026 |
| LPC (20:0) | -0.004 | 0.001 | 0.0051 |
| LPC (20:1) | -0.006 | 0.001 | 6.48E-15 |
| LPC (20:2) | -0.002 | 0.002 | 0.0002 |
| LPC (20:3) | 0.008 | 0.002 | 5.45E-21 |
| LPC (20:4) | -0.003 | 0.002 | 2.78E-14 |
| LPC (20:5) | 0.000 | 0.002 | 1.73E-18 |
| LPC (22:4) | 0.004 | 0.002 | 6.16E-10 |
| LPC (22:5) | -0.003 | 0.002 | 4.70E-15 |
| LPC (22:6) | -0.003 | 0.002 | 1.23E-11 |
| LPC (o-16:0) | -0.004 | 0.002 | 1.80E-27 |
| LPC (p-16:0) or LPC (o-16:1) | -0.006 | 0.002 | 1.49E-06 |
| LPC (p-18:0) or LPC (o-18:1) | -0.006 | 0.002 | 2.74E-47 |
| LPE (16:0) | 0.006 | 0.002 | 6.92E-11 |
| LPE (18:0) | 0.006 | 0.001 | 1.13E-23 |
| LPE (18:2) | 0.006 | 0.002 | 1.07E-08 |
| PC (30:0) | 0.016 | 0.002 | 0.0406 |
| PC (32:2) | 0.016 | 0.002 | 0.0055 |
| PC (33:2) | 0.005 | 0.002 | 0.0335 |
| PC (34:0) | 0.007 | 0.001 | 0.0054 |
| PC (34:1) | 0.014 | 0.002 | 0.0227 |
| PC (34:2) | 0.008 | 0.002 | 6.11E-15 |
| PC (34:3) | 0.009 | 0.002 | 0.0188 |
| PC (34:3) A | 0.001 | 0.002 | 0.0344 |
| PC (34:3) B | 0.007 | 0.002 | 2.30E-13 |
| PC (34:4) | 0.017 | 0.001 | 0.0160 |
| PC (35:2) | -0.004 | 0.002 | 0.0160 |
| PC (35:3) | 0.008 | 0.002 | 3.32E-06 |
| PC (35:4) | 0.005 | 0.002 | 1.08E-06 |
| PC (36:2) | 0.007 | 0.002 | 0.0223 |
| PC (36:3) A | 0.007 | 0.002 | 1.93E-06 |
| PC (36:5) A | -0.003 | 0.002 | 2.54E-67 |
| PC (36:5) B | 0.005 | 0.002 | 1.13E-48 |
| PC (36:5) C | 0.002 | 0.002 | 8.05E-36 |
| PC (36:6) | 0.011 | 0.002 | 6.43E-42 |
| PC (37:2) | -0.002 | 0.002 | 2.17E-64 |
| PC (37:3) | 0.009 | 0.002 | 3.82E-63 |
| PC (37:4) | -0.003 | 0.002 | 5.86E-43 |
| PC (37:5) | 0.002 | 0.002 | 1.74E-98 |
| PC (37:6) | 0.001 | 0.002 | 1.01E-96 |
| PC (38:2) | 0.011 | 0.002 | 1.98E-87 |
| PC (38:3) | 0.015 | 0.002 | 3.52E-112 |
| PC (38:4) | 0.004 | 0.002 | 3.35E-113 |
| PC (38:4) A | 0.005 | 0.002 | 1.51E-106 |
| PC (38:4) B | 0.010 | 0.002 | 8.27E-101 |
| PC (38:5) A | -0.001 | 0.002 | 3.86E-82 |
| PC (38:5) B | 0.005 | 0.002 | 3.64E-98 |
| PC (38:6) | 0.004 | 0.002 | 1.05E-129 |
| PC (38:6) A | 0.004 | 0.002 | 1.80E-128 |
| PC (38:6) B | 0.009 | 0.002 | 7.46E-106 |
| PC (38:6) C | 0.004 | 0.002 | 2.07E-73 |
| PC (38:7) | 0.000 | 0.002 | 3.32E-57 |
| PC (39:6) | -0.003 | 0.002 | 3.66E-109 |
| PC (40:4) | 0.017 | 0.002 | 2.24E-122 |
| PC (40:5) A | 0.008 | 0.002 | 4.45E-111 |
| PC (40:5) B | 0.013 | 0.002 | 1.22E-101 |
| PC (40:6) A | 0.001 | 0.002 | 2.38E-104 |
| PC (40:6) B | 0.006 | 0.002 | 5.95E-114 |
| PC (40:7) | -0.001 | 0.002 | 2.42E-136 |
| PC (40:8) | 0.002 | 0.002 | 2.17E-114 |
| PC (42:10) | 0.002 | 0.002 | 3.79E-79 |
| PC (42:5) | 0.009 | 0.002 | 9.34E-69 |
| PC (42:6) | 0.015 | 0.002 | 1.30E-64 |
| PC (42:7) | -0.004 | 0.002 | 2.82E-113 |
| PC (o-32:0) | -0.016 | 0.002 | 4.14E-120 |
| PC (o-34:0) | -0.006 | 0.001 | 5.06E-91 |
| PC (p-32:0) or PC (o-32:1) | -0.016 | 0.002 | 2.02E-59 |
| PC (p-32:1) or PC (o-32:2) | -0.004 | 0.002 | 2.61E-48 |
| PC (p-34:0) or PC (o-34:1) | -0.021 | 0.001 | 2.03E-87 |
| PC (p-34:1) or PC (o-34:2) A | -0.013 | 0.002 | 4.59E-161 |
| PC (p-34:1) or PC (o-34:2) B | -0.026 | 0.001 | 3.61E-50 |
| PC (p-34:2) or PC (o-34:3) | -0.019 | 0.002 | 9.70E-83 |
| PC (p-36:1) or PC (o-36:2) | -0.015 | 0.002 | 2.02E-49 |
| PC (p-36:1) or PC (o-36:2) A | -0.018 | 0.001 | 7.27E-45 |
| PC (p-36:2) or PC (o-36:3) | -0.013 | 0.002 | 4.72E-47 |
| PC (p-36:3) or PC (o-36:4) | -0.006 | 0.002 | 3.44E-121 |
| PC (p-36:4) or PC (o-36:5) | -0.011 | 0.002 | 2.01E-96 |
| PC (p-38:2) or PC (o-38:3) | <0.001 | 0.002 | 3.70E-78 |
| PC (p-38:3) or PC (o-38:4) | -0.009 | 0.002 | 1.43E-59 |
| PC (p-38:3) or PC (o-38:4) B | -0.008 | 0.002 | 1.69E-36 |
| PC (p-38:4) or PC (o-38:5) A | -0.014 | 0.002 | 1.12E-75 |
| PC (p-38:4) or PC (o-38:5) B | -0.015 | 0.002 | 4.29E-130 |
| PC (p-38:5) or PC (o-38:6) | -0.015 | 0.002 | 4.67E-136 |
| PC (p-38:5) or PC (o-38:6) A | -0.003 | 0.002 | 7.44E-45 |
| PC (p-38:6) or PC (o-38:7) | -0.006 | 0.002 | 1.58E-30 |
| PC (p-40:1) or PC (o-40:2) | -0.007 | 0.002 | 2.46E-17 |
| PC (p-40:3) or PC (o-40:4) | -0.011 | 0.002 | 1.90E-66 |
| PC (p-40:4) or PC (o-40:5) | -0.011 | 0.002 | 5.09E-17 |
| PC (p-40:5) or PC (o-40:6) | -0.005 | 0.002 | 2.17E-59 |
| PC (p-40:6) or PC (o-40:7) A | -0.004 | 0.002 | 5.00E-08 |
| PC (p-40:6) or PC (o-40:7) B | -0.012 | 0.002 | 1.09E-34 |
| PC (p-40:7) or PC (o-40:8) | -0.006 | 0.002 | 2.48E-19 |
| PC (p-42:2) or PC (o-42:3) | -0.015 | 0.002 | 9.51E-22 |
| PC (p-42:3) or PC (o-42:4) | -0.013 | 0.002 | 1.08E-127 |
| PC (p-42:4) or PC (o-42:5) | -0.014 | 0.002 | 9.51E-88 |
| PC (p-42:5) or PC (o-42:6) | -0.016 | 0.002 | 5.36E-54 |
| PC (p-42:5) or PC (o-42:6) A | -0.010 | 0.002 | 5.44E-108 |
| PC (p-44:4) or PC (o-44:5) | -0.013 | 0.002 | 7.91E-120 |
| PC (p-44:5) or PC (o-44:6) | -0.011 | 0.002 | 7.01E-89 |
| PE (32:1) | 0.017 | 0.002 | 6.06E-42 |
| PE (34:1) | 0.018 | 0.001 | 4.21E-58 |
| PE (34:2) | 0.017 | 0.002 | 6.22E-29 |
| PE (34:3) | 0.008 | 0.002 | 6.03E-42 |
| PE (36:1) | 0.021 | 0.001 | 1.33E-23 |
| PE (36:2) | 0.020 | 0.001 | 1.06E-32 |
| PE (36:3) | 0.013 | 0.002 | 5.09E-17 |
| PE (36:4) | 0.010 | 0.001 | 3.51E-21 |
| PE (38:2) | 0.007 | 0.002 | 5.34E-10 |
| PE (38:4) | 0.013 | 0.001 | 9.49E-35 |
| PE (38:4) A | 0.007 | 0.002 | 8.31E-116 |
| PE (38:4) B | 0.013 | 0.001 | 7.49E-89 |
| PE (38:6) | 0.009 | 0.001 | 0.0006 |
| PE (38:7) | 0.001 | 0.002 | 6.46E-76 |
| PE (40:6) | 0.002 | 0.002 | 2.37E-63 |
| PE (40:7) | 0.002 | 0.002 | 1.97E-59 |
| PE (40:8) | 0.000 | 0.002 | 1.07E-37 |
| PE (44:3) | 0.002 | 0.002 | 6.51E-52 |
| PE (p-34:1) or PE (o-34:2) | <0.001 | 0.002 | 3.08E-12 |
| PE (p-34:2) or PE (o-34:3) | 0.000 | 0.002 | 3.57E-12 |
| PE (p-36:1) or PE (o-36:2) | -0.002 | 0.002 | 5.98E-50 |
| PE (p-36:2) or PE (o-36:3) | -0.004 | 0.002 | 7.58E-49 |
| PE (p-36:4) or PE (o-36:5) | 0.001 | 0.001 | 8.85E-06 |
| PE (p-36:5) or PE (o-36:6) | 0.003 | 0.002 | 5.22E-50 |
| PE (p-38:2) or PE (o-38:3) | -0.003 | 0.002 | 1.01E-46 |
| PE (p-38:3) or PE (o-38:4) | -0.002 | 0.002 | 1.58E-33 |
| PE (p-38:4) or PE (o-38:5) | <0.001 | 0.002 | 6.55E-22 |
| PE (p-38:5) or PE (o-38:6) | -0.002 | 0.001 | 6.46E-23 |
| PE (p-38:6) or PE (o-38:7) | 0.001 | 0.002 | 3.97E-18 |
| PE (p-40:4) or PE (o-40:5) | 0.000 | 0.001 | 7.97E-06 |
| PE (p-40:4) or PE (o-40:5) A | 0.000 | 0.001 | 4.56E-13 |
| PE (p-40:4) or PE (o-40:5) B | 0.001 | 0.001 | 1.94E-06 |
| PE (p-40:5) or PE (o-40:6) | -0.001 | 0.002 | 2.27E-19 |
| PE (p-40:6) or PE (o-40:7) | -0.004 | 0.001 | 1.12E-14 |
| PG (34:2) A | 0.017 | 0.002 | 4.50E-29 |
| PG (34:2) B | 0.001 | 0.002 | 2.51E-36 |
| PG (36:4) | 0.005 | 0.002 | 2.34E-26 |
| PG (38:7) | -0.001 | 0.002 | 1.51E-38 |
| PG (40:7) | 0.001 | 0.002 | 1.29E-17 |
| PG (40:8) | 0.001 | 0.002 | 1.16E-21 |
| PG (44:12) | -0.003 | 0.002 | 2.10E-29 |
| PI (32:1) | 0.020 | 0.001 | 9.07E-15 |
| PI (34:1) | 0.017 | 0.002 | 1.53E-10 |
| PI (34:2) | 0.017 | 0.002 | 3.76E-10 |
| PI (36:1) | 0.010 | 0.002 | 3.95E-14 |
| PI (38:3) | 0.013 | 0.002 | 0.0251 |
| SM (d30:1) | 0.004 | 0.002 | 0.0461 |
| SM (d32:2) | 0.001 | 0.001 | 0.0110 |
| SM (d36:3) | -0.004 | 0.002 | 0.0060 |
| SM (d38:2) | -0.012 | 0.002 | 0.0023 |
| SM (d39:2) | -0.013 | 0.002 | 0.0283 |
| SM (d40:2) A | 0.001 | 0.002 | 0.0033 |
| SM (d40:2) B | -0.005 | 0.002 | 0.0495 |
| SM (d40:3) | -0.010 | 0.002 | 1.05E-05 |
| SM (d41:1) | 0.004 | 0.002 | 0.0112 |
| SM (d41:2) A | -0.002 | 0.002 | 0.0060 |
| SM (d42:0) | 0.008 | 0.002 | 1.21E-17 |
| SM (d42:1) | 0.002 | 0.002 | 2.66E-07 |
| SM (d42:2) B | -0.006 | 0.002 | 8.04E-05 |
| SM (d42:3) | -0.007 | 0.001 | 0.0002 |
| SM (d43:1) | 0.002 | 0.002 | 0.0003 |
| SM (d43:2) B | 0.001 | 0.002 | 0.0027 |
| SM (d44:2) | -0.003 | 0.002 | 2.50E-19 |
| TG (14:0/14:0/14:0) | 0.026 | 0.001 | 8.28E-09 |
| TG (40:0) | 0.023 | 0.002 | 0.0050 |
| TG (40:1) | 0.019 | 0.002 | 2.39E-07 |
| TG (42:0) | 0.021 | 0.002 | 5.20E-18 |
| TG (42:1) | 0.025 | 0.001 | 9.16E-07 |
| TG (42:3) | 0.020 | 0.001 | 5.25E-05 |
| TG (44:1) | 0.029 | 0.001 | 0.0257 |
| TG (44:2) | 0.028 | 0.001 | 0.0079 |
| TG (46:0) | 0.030 | 0.001 | 3.62E-18 |
| TG (46:1) | 0.030 | 0.001 | 2.85E-05 |
| TG (46:2) | 0.030 | 0.001 | 8.31E-06 |
| TG (46:3) | 0.029 | 0.001 | 2.44E-14 |
| TG (46:4) A | 0.028 | 0.001 | 0.0015 |
| TG (48:0) | 0.028 | 0.001 | 0.0058 |
| TG (48:2) | 0.031 | 0.001 | 0.0033 |
| TG (48:5) | 0.024 | 0.001 | 7.73E-12 |
| TG (49:0) | 0.029 | 0.001 | 6.81E-22 |
| TG (49:1) | 0.030 | 0.001 | 0.0110 |
| TG (49:3) | 0.029 | 0.001 | 0.0089 |
| TG (50:0) | 0.029 | 0.001 | 0.0141 |
| TG (50:1) | 0.029 | 0.001 | 3.97E-25 |
| TG (50:2) | 0.030 | 0.001 | 7.80E-07 |
| TG (50:3) | 0.029 | 0.001 | 1.47E-15 |
| TG (50:5) | 0.025 | 0.001 | 5.79E-05 |
| TG (51:1) | 0.029 | 0.001 | 8.16E-24 |
| TG (51:2) | 0.029 | 0.001 | 1.74E-23 |
| TG (51:3) | 0.027 | 0.001 | 8.57E-47 |
| TG (51:4) | 0.024 | 0.001 | 2.68E-15 |
| TG (51:5) | 0.022 | 0.002 | 6.53E-81 |
| TG (52:0) | 0.028 | 0.001 | 1.42E-33 |
| TG (52:1) | 0.032 | 0.001 | 2.15E-21 |
| TG (52:2) | 0.022 | 0.001 | 5.91E-16 |
| TG (52:3) | 0.026 | 0.001 | 0.0007 |
| TG (52:4) | 0.021 | 0.001 | 9.69E-12 |
| TG (52:5) | 0.021 | 0.001 | 3.21E-08 |
| TG (52:6) | 0.022 | 0.001 | 2.94E-16 |
| TG (53:1) | 0.030 | 0.001 | 1.52E-23 |
| TG (53:2) | 0.027 | 0.001 | 1.48E-19 |
| TG (53:3) | 0.025 | 0.001 | 1.53E-25 |
| TG (53:4) | 0.023 | 0.001 | 1.93E-28 |
| TG (53:5) | 0.019 | 0.002 | 3.87E-37 |
| TG (54:0) | 0.026 | 0.001 | 2.05E-45 |
| TG (54:1) | 0.031 | 0.001 | 1.50E-16 |
| TG (54:2) | 0.030 | 0.001 | 2.27E-05 |
| TG (54:3) | 0.021 | 0.001 | 6.18E-05 |
| TG (54:4) | 0.018 | 0.002 | 2.68E-23 |
| TG (55:3) | 0.022 | 0.001 | 0.0044 |
| TG (56:2) | 0.030 | 0.001 | 2.63E-25 |
| TG (56:4) | 0.021 | 0.002 | 0.0116 |
| TG (56:7) B | 0.017 | 0.001 | 1.02E-41 |
| TG (56:8) A | 0.014 | 0.002 | 3.25E-28 |
| TG (56:8) B | 0.014 | 0.002 | 9.72E-30 |
| TG (56:9) | 0.010 | 0.002 | 1.03E-10 |
| TG (57:1) | 0.019 | 0.002 | 1.72E-07 |
| TG (57:2) | 0.030 | 0.001 | 1.04E-06 |
| TG (58:1) | 0.027 | 0.001 | 1.79E-32 |
| TG (58:10) | 0.006 | 0.002 | 1.29E-15 |
| TG (58:2) | 0.026 | 0.001 | 6.09E-11 |
| TG (58:3) | 0.024 | 0.001 | 0.0003 |
| TG (58:4) A | 0.024 | 0.001 | 5.57E-09 |
| TG (58:5) | 0.020 | 0.002 | 1.50E-06 |
| TG (58:6) | 0.022 | 0.001 | 1.79E-11 |
| TG (58:8) | 0.011 | 0.002 | 1.09E-15 |
| TG (58:9) | 0.011 | 0.002 | 8.45E-06 |
| TG (60:11) | 0.007 | 0.002 | 0.0240 |
| TG (60:2) | 0.022 | 0.001 | 1.65E-14 |
| TG (60:3) | 0.021 | 0.001 | 2.79E-16 |
| TG (60:4) | 0.019 | 0.002 | 0.0018 |
| TG (60:6) | 0.016 | 0.002 | 1.73E-09 |
| TG (62:3) | 0.016 | 0.002 | 3.09E-07 |
| TG (62:4) | 0.014 | 0.002 | 0.0007 |
| *Adjusted for age, sex, BMI, center and family relationship.  † Metabolite values were rank-inverse transferred.  ‡ P-values were FDR corrected to account for multiple comparisons. Results are shown for significant metabolites (FDR-adjusted P < 0.05).  Abbreviations: *SE=* Standard Error*,* *CE=* cholesteryl ester*, DG=* diglycerides*, FA=* Fatty acid*, LPC=* lysophosphatidylcholine*, LPE=* lysophosphoethanolamine*, PC=* phosphatidylcholine, *PE=* phosphoethanolamine*, PG=* prostaglandin, *PI=* phosphatidylinositol*, PS=* phosphatidylserine*, SM=* sphingomyelin*, TG=* triglycerides*.* | | | |

| Supplementary Table 2- Factors identified from exploratory factor analysis following principal components analysis (PCA) in the Lipid Lowering Drugs and Diet Network study of lipidomics. Only metabolites with a factor **loading ≥ 0.5** were reported as composing a given factor. | | | | | | | |
| --- | --- | --- | --- | --- | --- | --- | --- |
| Factor 1 | |  | Factor 2 | |  | Factor 3 | |
| Metabolites | loadings |  | Metabolites | loadings |  | Metabolites | loadings |
| TG (52:4) | 0.96 |  | SM (d42:3) | 0.80 |  | PC (30:1) | 0.88 |
| TG (53:4) | 0.95 |  | SM (d42:2) A | 0.77 |  | PC (30:0) | 0.87 |
| TG (52:3) | 0.94 |  | SM (d34:1) | 0.77 |  | PC (28:0) | 0.87 |
| TG (52:5) | 0.94 |  | SM (d38:1) | 0.76 |  | TG (44:0) | 0.86 |
| DG (36:3) | 0.93 |  | SM (d34:2) | 0.76 |  | PC (32:3) | 0.86 |
| TG (51:4) | 0.92 |  | SM (d41:2) B | 0.76 |  | TG (46:0) | 0.85 |
| DG (36:4) | 0.91 |  | PC (p-38:5) or PC (o-38:6) A | 0.75 |  | TG (44:1) | 0.85 |
| TG (54:4) | 0.90 |  | SM (d33:1) | 0.75 |  | TG (46:1) | 0.84 |
| TG (53:5) | 0.90 |  | SM (d40:1) | 0.72 |  | TG (48:1) | 0.81 |
| TG (50:4) | 0.90 |  | SM (d41:1) | 0.72 |  | TG (49:0) | 0.80 |
| TG (53:3) | 0.89 |  | SM (d41:2) | 0.70 |  | TG (14:0/14:0/14:0) | 0.79 |
| TG (51:3) | 0.87 |  | SM (d32:1) | 0.69 |  | TG (42:1) | 0.78 |
| TG (54:5) A | 0.87 |  | SM (d39:1) | 0.68 |  | TG (48:0) | 0.77 |
| TG (56:6) | 0.85 |  | PC (37:5) | 0.67 |  | TG (46:2) | 0.77 |
| TG (52:6) | 0.85 |  | PC (p-36:1) or PC (o-36:2) A | 0.67 |  | TG (44:2) | 0.76 |
| TG (50:3) | 0.85 |  | SM (d40:2) A | 0.66 |  | TG (49:1) | 0.74 |
| TG (54:3) | 0.84 |  | PE (p-40:7) or PE (o-40:8) | 0.66 |  | TG (48:2) | 0.74 |
| TG (50:5) | 0.83 |  | SM (d36:2) | 0.65 |  | TG (40:0) | 0.72 |
| TG (54:6) A | 0.83 |  | SM (d44:2) | 0.65 |  | PI (32:1) | 0.71 |
| TG (56:5) A | 0.83 |  | PE (p-40:6) or PE (o-40:7) | 0.64 |  | TG (51:1) | 0.70 |
| TG (56:7) A | 0.83 |  | SM (d42:1) | 0.64 |  | TG (46:3) | 0.70 |
| TG (51:5) | 0.81 |  | PC (p-32:1) or PC (o-32:2) | 0.64 |  | TG (49:2) | 0.68 |
| TG (56:3) | 0.81 |  | Ceramide (d34:2) | 0.63 |  | PC (31:1) | 0.67 |
| TG (54:6) B | 0.81 |  | PE (p-38:5) or PE (o-38:6) | 0.63 |  | TG (42:0) | 0.67 |
| DG (36:2) | 0.80 |  | GlcCer (d38:1) | 0.62 |  | PI (34:1) | 0.65 |
| TG (56:4) | 0.78 |  | PC (38:4) A | 0.62 |  | PC (32:2) | 0.65 |
| DG (38:6) A | 0.78 |  | PC (42:10) | 0.61 |  | PC (34:4) | 0.65 |
| TG (58:6) | 0.77 |  | Ceramide (d34:1) | 0.61 |  | TG (42:2) | 0.65 |
| TG (54:2) | 0.77 |  | PC (38:6) A | 0.61 |  | PC (33:0) | 0.65 |
| DG (38:6) B | 0.77 |  | SM (d32:0) | 0.61 |  | PC (33:1) | 0.64 |
| TG (58:4) A | 0.75 |  | PC (p-38:6) or PC (o-38:7) | 0.60 |  | TG (50:1) | 0.64 |
| TG (53:2) | 0.75 |  | PC (p-32:0) or PC (o-32:1) | 0.60 |  | DG (32:1) | 0.64 |
| TG (58:3) | 0.74 |  | PE (p-36:5) or PE (o-36:6) | 0.58 |  | LPC (14:0) | 0.64 |
| TG (54:7) B | 0.74 |  | PC (p-34:1) or PC (o-34:2) B | 0.58 |  | TG (48:3) | 0.63 |
| TG (56:2) | 0.74 |  | PC (38:6) | 0.57 |  | PC (32:1) | 0.63 |
| TG (49:3) | 0.73 |  | PC (39:6) | 0.57 |  | TG (50:2) | 0.63 |
| DG (38:5) | 0.73 |  | PE (p-36:4) or PE (o-36:5) | 0.57 |  | TG (50:0) | 0.63 |
| TG (56:5) B | 0.72 |  | PI (38:4) | 0.56 |  | TG (53:1) | 0.63 |
| TG (51:2) | 0.72 |  | PC (36:3) A | 0.56 |  | PC (31:0) | 0.62 |
| TG (55:3) | 0.72 |  | CE (18:1) | 0.56 |  | TG (52:1) | 0.62 |
| TG (54:5) B | 0.72 |  | SM (d43:1) | 0.56 |  | PI (36:4) | 0.62 |
| DG (34:2) | 0.72 |  | PC (p-36:4) or PC (o-36:5) | 0.56 |  | PE (32:1) | 0.60 |
| TG (57:2) | 0.71 |  | Ceramide (d32:1) | 0.56 |  | PC (36:1) | 0.60 |
| TG (60:4) | 0.70 |  | CE (20:5) | 0.55 |  | PC (34:1) | 0.60 |
| TG (59:3) | 0.70 |  | Ceramide (d33:1) | 0.55 |  | TG (49:3) | 0.60 |
| TG (48:3) | 0.70 |  | PE (38:4) A | 0.55 |  | TG (55:1) | 0.59 |
| TG (50:2) | 0.70 |  | SM (d32:2) | 0.55 |  | TG (40:1) | 0.58 |
| TG (48:4) | 0.69 |  | GlcCer (d41:1) | 0.55 |  | TG (46:4) A | 0.57 |
| TG (54:7) A | 0.69 |  | PC (35:2) B | 0.55 |  | PE (34:1) | 0.57 |
| DG (34:3) | 0.69 |  | PE (p-38:6) or PE (o-38:7) | 0.55 |  | TG (52:0) | 0.56 |
| TG (55:2) | 0.69 |  | CE (22:6) | 0.54 |  | TG (51:2) | 0.55 |
| TG (56:7) B | 0.68 |  | PC (o-34:0) | 0.54 |  | TG (54:1) | 0.55 |
| TG (50:6) | 0.68 |  | CE (18:2) | 0.54 |  | SM (d30:1) | 0.54 |
| DG (34:1) | 0.68 |  | PC (40:8) | 0.54 |  | PC (38:4) B | 0.53 |
| TG (58:2) | 0.67 |  | PC (38:4) B | 0.54 |  | PC (36:4) C | 0.53 |
| TG (52:1) | 0.67 |  | CE (20:4) | 0.54 |  | DG (34:1) | 0.52 |
| DG (36:5) | 0.66 |  | SM (d39:2) | 0.54 |  |  |  |
| TG (60:3) | 0.66 |  | GlcCer (d42:2) | 0.53 |  |  |  |
| TG (54:8) B | 0.66 |  | PC (32:0) | 0.53 |  |  |  |
| TG (49:2) | 0.65 |  | PE (p-34:1) or PE (o-34:2) | 0.53 |  |  |  |
| TG (52:2) | 0.65 |  | PC (36:4) B | 0.53 |  |  |  |
| TG (56:8) B | 0.65 |  | PC (34:0) | 0.53 |  |  |  |
| TG (48:2) | 0.64 |  | PC (42:7) | 0.52 |  |  |  |
| TG (56:1) | 0.64 |  | PC (38:4) | 0.52 |  |  |  |
| TG (58:5) | 0.64 |  | PC (38:6) C | 0.52 |  |  |  |
| TG (56:8) A | 0.64 |  | PE (p-40:4) or PE (o-40:5) | 0.52 |  |  |  |
| TG (54:1) | 0.63 |  | SM (d36:1) | 0.52 |  |  |  |
| TG (58:1) | 0.62 |  | PC (31:0) | 0.52 |  |  |  |
| TG (56:9) | 0.62 |  | PC (38:7) | 0.52 |  |  |  |
| TG (60:2) | 0.62 |  | GlcCer (d42:1) | 0.52 |  |  |  |
| TG (55:1) | 0.62 |  | PC (p-38:4) or PC (o-38:5) B | 0.52 |  |  |  |
| DG (40:7) | 0.62 |  | Ceramide (d44:1) | 0.51 |  |  |  |
| TG (54:8) A | 0.61 |  | Lactosylceramide (d18:1/24:1(15Z)) | 0.51 |  |  |  |
| TG (48:5) | 0.61 |  | GlcCer (d40:1) | 0.51 |  |  |  |
| TG (59:2) | 0.61 |  | PC (35:3) | 0.51 |  |  |  |
| TG (46:3) | 0.60 |  | PC (38:5) A | 0.51 |  |  |  |
| TG (53:1) | 0.60 |  | PC (37:2) | 0.51 |  |  |  |
| TG (58:9) | 0.59 |  |  |  |  |  |  |
| TG (49:1) | 0.58 |  |  |  |  |  |  |
| TG (51:1) | 0.58 |  |  |  |  |  |  |
| TG (62:4) | 0.58 |  |  |  |  |  |  |
| TG (46:4) A | 0.57 |  |  |  |  |  |  |
| Ceramide (d40:1) | 0.57 |  |  |  |  |  |  |
| TG (50:1) | 0.57 |  |  |  |  |  |  |
| DG (40:6) | 0.57 |  |  |  |  |  |  |
| TG (58:8) | 0.56 |  |  |  |  |  |  |
| TG (46:2) | 0.56 |  |  |  |  |  |  |
| TG (48:1) | 0.55 |  |  |  |  |  |  |
| TG (62:3) | 0.55 |  |  |  |  |  |  |
| PE (36:2) | 0.54 |  |  |  |  |  |  |
| DG (32:1) | 0.53 |  |  |  |  |  |  |
| TG (50:0) | 0.52 |  |  |  |  |  |  |
| Ceramide (d18:1/23:0) | 0.52 |  |  |  |  |  |  |
| Ceramide (d38:1) | 0.51 |  |  |  |  |  |  |
| Ceramide (d40:2) | 0.51 |  |  |  |  |  |  |
| Ceramide (d42:1) | 0.50 |  |  |  |  |  |  |
| TG (46:1) | 0.50 |  |  |  |  |  |  |
| Abbreviations: *TG=* triglycerides*, DG=*diglycerides*, PE=*phosphoethanolamine*, SM=* sphingomyelin*, PC=*phosphatidylcholine, *PI=*phosphatidylinositol. | | | | | | | |

| Supplementary Table 3- Results of associations^*^ between homeostatic model assessment-insulin resistance and plasma lipids in the HAPI Heart study | | | | |
| --- | --- | --- | --- | --- |
| **Metabolites**^†^ | **β** | **SE** | **FDR-corrected P-value**^‡^ | |
| CE (16:0) | -0.141 | 0.038 | 0.0009 | |
| **CE (18:1)** | **-0.177** | **0.036** | **9.70E-06** | |
| **CE (18:2)** | **-0.122** | **0.038** | **0.0043** | |
| **CE (20:3)** | **-0.148** | **0.034** | **9.17E-05** | |
| **CE (20:4)** | **-0.114** | **0.039** | **0.0086** | |
| **CE (22:6)** | **-0.091** | **0.039** | **0.0387** | |
| **Ceramide (d34:2)** | **-0.085** | **0.036** | **0.0378** | |
| **Ceramide (d40:0)** | **0.113** | **0.035** | **0.0033** | |
| **Ceramide (d42:0)** | **0.102** | **0.036** | **0.0128** | |
| **DG (32:1)** | **0.158** | **0.028** | **2.90E-07** | |
| **DG (34:1)** | **0.184** | **0.031** | **1.74E-07** | |
| **DG (34:2)** | **0.228** | **0.037** | **6.03E-08** | |
| **DG (36:2)** | **0.125** | **0.034** | **0.0009** | |
| **DG (36:3)** | **0.097** | **0.034** | **0.0123** | |
| DG (36:4) B | 0.163 | 0.035 | 2.05E-05 | |
| **DG (38:5)** | **0.184** | **0.036** | **4.80E-06** | |
| **FA (14:1) (physeteric acid)** | **-0.068** | **0.027** | **0.0233** | |
| GlcCer (d34:1) | -0.106 | 0.039 | 0.0162 | |
| **GlcCer (d42:2)** | **-0.102** | **0.040** | **0.0233** | |
| **LPC (18:1)** | **-0.112** | **0.037** | **0.0067** | |
| **LPC (18:2)** | **-0.081** | **0.033** | **0.0291** | |
| **LPC (20:1)** | **-0.149** | **0.035** | **0.0001** | |
| **LPC (20:2)** | **-0.139** | **0.039** | **0.0012** | |
| **LPC (22:5)** | **-0.102** | **0.040** | **0.0230** | |
| PC (32:1) | 0.152 | 0.035 | 0.0001 | |
| PC (34:1e) | -0.104 | 0.040 | 0.0201 | |
| PC (34:3) C | 0.093 | 0.038 | 0.0309 | |
| **PC (34:4)** | **0.088** | **0.036** | **0.0319** | |
| PC (35:1) | -0.131 | 0.037 | 0.0016 | |
| PC (35:2) A | -0.145 | 0.039 | 0.0008 | |
| PC (36:1) | 0.104 | 0.038 | 0.0160 | |
| PC (36:3) B | 0.107 | 0.035 | 0.0068 | |
| PC (36:3e) | -0.119 | 0.040 | 0.0082 | |
| PC (36:4) C | 0.099 | 0.039 | 0.0258 | |
| **PC (37:3)** | **-0.093** | **0.039** | **0.0345** | |
| **PC (37:4)** | **-0.095** | **0.038** | **0.0281** | |
| PC (37:5e) | -0.153 | 0.039 | 0.0004 | |
| PC (37:5e).1 | -0.182 | 0.040 | 3.79E-05 | |
| PC (38:3) | 0.176 | 0.035 | 5.78E-06 | |
| **PC (38:4) B** | **0.095** | **0.042** | **0.0420** | |
| **PC (38:5) B** | **0.089** | **0.038** | **0.0400** | |
| **PC (40:4)** | **0.171** | **0.038** | **5.31E-05** | |
| **PC (40:5) A** | **0.110** | **0.039** | **0.0122** | |
| **PC (40:6) B** | **0.100** | **0.038** | **0.0196** | |
| PC (40:6e) | -0.122 | 0.041 | 0.0084 | |
| PC (40:7) B | -0.090 | 0.039 | 0.0395 | |
| **PC (42:5)** | **0.105** | **0.039** | **0.0184** | |
| **PC (42:6)** | **0.135** | **0.037** | **0.0010** | |
| **PC (o-32:0)** | **-0.181** | **0.035** | **2.45E-06** | |
| **PC (o-34:0)** | **-0.156** | **0.040** | **0.0004** | |
| **PC (p-32:0) or PC (o-32:1)** | **-0.221** | **0.036** | **4.60E-08** | |
| **PC (p-34:0) or PC (o-34:1)** | **-0.244** | **0.034** | **3.85E-10** | |
| **PC (p-34:1) or PC (o-34:2) A** | **-0.137** | **0.037** | **0.0009** | |
| **PC (p-34:1) or PC (o-34:2) B** | **-0.302** | **0.032** | **6.01E-17** | |
| **PC (p-34:2) or PC (o-34:3)** | **-0.252** | **0.035** | **2.28E-10** | |
| **PC (p-36:1) or PC (o-36:2) A** | **-0.190** | **0.039** | **1.31E-05** | |
| PC (p-36:1) or PC (o-36:2) B | -0.250 | 0.035 | 2.84E-10 | |
| **PC (p-36:2) or PC (o-36:3)** | **-0.177** | **0.036** | **1.13E-05** | |
| **PC (p-36:4) or PC (o-36:5)** | **-0.172** | **0.038** | **4.84E-05** | |
| **PC (p-38:2) or PC (o-38:3)** | **-0.109** | **0.042** | **0.0217** | |
| PC (p-38:3) or PC (o-38:4) A | -0.098 | 0.041 | 0.0336 | |
| **PC (p-38:4) or PC (o-38:5) A** | **-0.136** | **0.038** | **0.0013** | |
| **PC (p-38:4) or PC (o-38:5) B** | **-0.218** | **0.036** | **8.86E-08** | |
| **PC (p-38:6) or PC (o-38:7)** | **-0.094** | **0.040** | **0.0384** | |
| **PC (p-40:1) or PC (o-40:2)** | **-0.115** | **0.039** | **0.0082** | |
| **PC (p-40:3) or PC (o-40:4)** | **-0.157** | **0.037** | **0.0001** | |
| **PC (p-40:4) or PC (o-40:5)** | **-0.198** | **0.037** | **1.54E-06** | |
| **PC (p-40:6) or PC (o-40:7) B** | **-0.186** | **0.038** | **1.28E-05** | |
| **PC (p-42:2) or PC (o-42:3)** | **-0.173** | **0.040** | **7.84E-05** | |
| **PC (p-42:3) or PC (o-42:4)** | **-0.111** | **0.040** | **0.0123** | |
| **PC (p-42:4) or PC (o-42:5)** | **-0.201** | **0.037** | **1.12E-06** | |
| **PC (p-42:5) or PC (o-42:6) A** | **-0.168** | **0.040** | **0.0001** | |
| **PC (p-42:5) or PC (o-42:6) B** | **-0.165** | **0.036** | **3.76E-05** | |
| **PC (p-44:4) or PC (o-44:5)** | **-0.187** | **0.036** | **3.09E-06** | |
| **PC (p-44:5) or PC (o-44:6)** | **-0.121** | **0.036** | **0.0028** | |
| **PE (34:1)** | **0.116** | **0.033** | **0.0017** | |
| **PE (34:2)** | **0.129** | **0.032** | **0.0003** | |
| **PE (36:1)** | **0.127** | **0.027** | **1.76E-05** | |
| **PE (36:2)** | **0.135** | **0.029** | **2.09E-05** | |
| **PE (36:3)** | **0.060** | **0.026** | **0.0400** | |
| **PE (36:4)** | **0.092** | **0.035** | **0.0196** | |
| PE (38:4) B | 0.084 | 0.038 | 0.0483 | |
| **PE (38:6)** | **0.077** | **0.034** | **0.0476** | |
| **PE (p-34:1) or PE (o-34:2)** | **-0.104** | **0.040** | **0.0230** | |
| **PE (p-38:2) or PE (o-38:3)** | **-0.093** | **0.039** | **0.0341** | |
| **PE (p-38:3) or PE (o-38:4)** | **-0.091** | **0.042** | **0.0547** | |
| **PE (p-38:4) or PE (o-38:5)** | **-0.098** | **0.041** | **0.0339** | |
| **PE (p-40:5) or PE (o-40:6)** | **-0.100** | **0.042** | **0.0348** | |
| **PE (p-40:6) or PE (o-40:7)** | **-0.123** | **0.040** | **0.0061** | |
| PI 36:2; PI 18:0-18:2; | 0.097 | 0.036 | 0.0172 | |
| PI 36:4; PI 16:0-20:4; | 0.199 | 0.034 | 1.74E-07 | |
| SM (d32:0) | -0.090 | 0.039 | 0.0384 | |
| SM (d32:1) | -0.091 | 0.039 | 0.0384 | |
| **SM (d32:2)** | **-0.085** | **0.030** | **0.0123** | |
| SM (d33:1) | -0.142 | 0.038 | 0.0009 | |
| SM (d34:0) | -0.140 | 0.037 | 0.0006 | |
| SM (d34:1) | -0.174 | 0.035 | 9.47E-06 | |
| SM (d34:2) | -0.092 | 0.033 | 0.0132 | |
| SM (d36:1) | -0.089 | 0.035 | 0.0246 | |
| **SM (d36:3)** | **-0.107** | **0.037** | **0.0094** | |
| SM (d37:1) | -0.133 | 0.036 | 0.0009 | |
| **SM (d38:2)** | **-0.171** | **0.033** | **2.43E-06** | |
| **SM (d39:2)** | **-0.180** | **0.034** | **1.54E-06** | |
| SM (d40:0) | 0.093 | 0.035 | 0.0172 | |
| **SM (d40:2) A** | **-0.108** | **0.042** | **0.0233** | |
| **SM (d40:2) B** | **-0.157** | **0.037** | **0.0001** | |
| **SM (d40:3)** | **-0.197** | **0.036** | **1.27E-06** | |
| **SM (d41:2) A** | **-0.159** | **0.038** | **0.0002** | |
| SM (d41:2) B | -0.152 | 0.039 | 0.0004 | |
| **SM (d42:0)** | **0.098** | **0.038** | **0.0230** | |
| SM (d42:2) | -0.116 | 0.041 | 0.0116 | |
| SM (d42:2) A | -0.150 | 0.038 | 0.0004 | |
| **SM (d42:2) B** | **-0.167** | **0.037** | **4.84E-05** | |
| **SM (d42:3)** | **-0.224** | **0.036** | **2.54E-08** | |
| SM (d43:2) | -0.133 | 0.038 | 0.0015 | |
| **SM (d44:2)** | **-0.122** | **0.038** | **0.0042** | |
| **TG (52:4)** | **0.199** | **0.035** | **2.51E-07** | |
| **TG (53:5)** | **0.118** | **0.035** | **0.0024** | |
| **TG (40:1)** | **0.057** | **0.024** | **0.0336** | |
| **TG (42:0)** | **0.080** | **0.024** | **0.0024** | |
| **TG (42:1)** | **0.082** | **0.024** | **0.0017** | |
| TG (42:2) | 0.090 | 0.024 | 0.0007 | |
| TG (44:0) | 0.087 | 0.023 | 0.0006 | |
| **TG (44:1)** | **0.101** | **0.024** | **0.0001** | |
| **TG (44:2)** | **0.116** | **0.024** | **1.50E-05** | |
| **TG (46:0)** | **0.092** | **0.024** | **0.0006** | |
| **TG (46:1)** | **0.131** | **0.024** | **8.49E-07** | |
| **TG (46:2)** | **0.136** | **0.025** | **7.46E-07** | |
| TG (46:3) A | 0.120 | 0.024 | 5.83E-06 | |
| TG (46:3) B | 0.119 | 0.025 | 1.32E-05 | |
| TG (46:4) B | 0.103 | 0.026 | 0.0003 | |
| **TG (48:0)** | **0.147** | **0.028** | **1.54E-06** | |
| TG (48:1) | 0.143 | 0.024 | 1.56E-07 | |
| TG (48:2) A | 0.167 | 0.027 | 4.55E-08 | |
| TG (48:2) B | 0.177 | 0.027 | 4.85E-09 | |
| TG (48:3) | 0.155 | 0.029 | 1.54E-06 | |
| TG (48:4) A | 0.175 | 0.030 | 2.13E-07 | |
| TG (48:4) B | 0.181 | 0.031 | 1.30E-07 | |
| **TG (49:0)** | **0.076** | **0.024** | **0.0050** | |
| **TG (49:1)** | **0.115** | **0.025** | **2.17E-05** | |
| TG (49:2) | 0.127 | 0.028 | 3.83E-05 | |
| **TG (49:3)** | **0.136** | **0.030** | **3.87E-05** | |
| **TG (50:1)** | **0.183** | **0.027** | **1.77E-09** | |
| **TG (50:2)** | **0.189** | **0.029** | **1.31E-08** | |
| TG (50:3) A | 0.173 | 0.033 | 2.29E-06 | |
| TG (50:3) B | 0.174 | 0.033 | 2.15E-06 | |
| TG (50:4) | 0.158 | 0.035 | 3.44E-05 | |
| TG (50:5) A | 0.173 | 0.037 | 2.05E-05 | |
| TG (50:5) B | 0.188 | 0.037 | 4.26E-06 | |
| **TG (51:1)** | **0.110** | **0.024** | **3.57E-05** | |
| **TG (51:2)** | **0.133** | **0.029** | **3.79E-05** | |
| **TG (51:3)** | **0.125** | **0.034** | **0.0009** | |
| **TG (51:4)** | **0.109** | **0.034** | **0.0041** | |
| **TG (51:5)** | **0.147** | **0.036** | **0.0003** | |
| TG (52:0) B | 0.118 | 0.034 | 0.0020 | |
| **TG (52:1)** | **0.150** | **0.024** | **3.79E-08** | |
| **TG (52:2)** | **0.163** | **0.034** | **1.28E-05** | |
| **TG (52:3)** | **0.142** | **0.035** | **0.0003** | |
| TG (52:5) A | 0.141 | 0.035 | 0.0003 | |
| TG (52:5) B | 0.143 | 0.038 | 0.0007 | |
| **TG (52:6)** | **0.217** | **0.037** | **1.94E-07** | |
| **TG (53:1)** | **0.098** | **0.023** | **0.0002** | |
| TG (53:2) A | 0.101 | 0.030 | 0.0022 | |
| TG (53:2) B | 0.104 | 0.030 | 0.0016 | |
| **TG (53:3)** | **0.110** | **0.033** | **0.0032** | |
| **TG (53:4)** | **0.125** | **0.036** | **0.0020** | |
| **TG (54:0)** | **0.084** | **0.034** | **0.0309** | |
| **TG (54:1)** | **0.123** | **0.024** | **2.45E-06** | |
| TG (54:2) A | 0.096 | 0.030 | 0.0042 | |
| TG (54:2) B | 0.138 | 0.026 | 1.47E-06 | |
| **TG (54:3)** | **0.095** | **0.035** | **0.0177** | |
| TG (54:5) B | 0.177 | 0.036 | 9.12E-06 | |
| TG (54:6) B | 0.101 | 0.036 | 0.0128 | |
| TG (54:6) C | 0.170 | 0.036 | 2.27E-05 | |
| TG (54:7) B | 0.173 | 0.038 | 4.84E-05 | |
| TG (55:1) | 0.091 | 0.024 | 0.0006 | |
| TG (55:2) | 0.076 | 0.025 | 0.0065 | |
| **TG (55:3)** | **0.081** | **0.030** | **0.0168** | |
| TG (56:1) | 0.145 | 0.028 | 2.61E-06 | |
| **TG (56:2)** | **0.141** | **0.029** | **8.44E-06** | |
| TG (56:3) | 0.128 | 0.030 | 0.0001 | |
| **TG (56:4)** | **0.100** | **0.035** | **0.0119** | |
| TG (56:5) A | 0.143 | 0.034 | 0.0001 | |
| TG (56:5) C | 0.103 | 0.035 | 0.0091 | |
| TG (56:6) A | 0.124 | 0.038 | 0.0032 | |
| TG (56:6) B | 0.105 | 0.036 | 0.0088 | |
| TG (56:7) A | 0.102 | 0.040 | 0.0255 | |
| **TG (56:7) B** | **0.127** | **0.036** | **0.0013** | |
| **TG (56:8) B** | **0.115** | **0.036** | **0.0039** | |
| **TG (56:9)** | **0.091** | **0.036** | **0.0263** | |
| **TG (57:2)** | **0.091** | **0.025** | **0.0013** | |
| **TG (58:1)** | **0.167** | **0.031** | **1.47E-06** | |
| **TG (58:10)** | **0.099** | **0.040** | **0.0301** | |
| **TG (58:2)** | **0.163** | **0.037** | **5.74E-05** | |
| **TG (58:3)** | **0.149** | **0.042** | **0.0015** | |
| **TG (58:5)** | **0.128** | **0.035** | **0.0010** | |
| **TG (58:6)** | **0.140** | **0.034** | **0.0002** | |
| **TG (58:9)** | **0.123** | **0.039** | **0.0048** | |
| TG (59:2) | 0.087 | 0.027 | 0.0047 | |
| TG (60:1) | 0.144 | 0.030 | 1.09E-05 | |
| **TG (60:2)** | **0.128** | **0.036** | **0.0014** | |
| *Adjusted for age, sex, BMI, center and family relationship.  † Metabolite values were rank-inverse transferred.  ‡ P-values were FDR corrected to account for multiple comparisons. Results are shown for significant metabolites (FDR-adjusted P < 0.05). Bolded values indicate metabolites that overlapped between the main and replication study.  Abbreviations: *SE=* Standard Error*,* *CE=* cholesteryl ester*, DG=* diglycerides*, FA=* Fatty acid*, LPC=* lysophosphatidylcholine*, LPE=* lysophosphoethanolamine*, PC=* phosphatidylcholine, *PE=* phosphoethanolamine*, PI=* phosphatidylinositol*, SM=* sphingomyelin*, TG=* triglycerides*.* | | | |  |
|  | | | | |

| **Supplementary Table 4- Characteristics^*^ of participants by quintile of the homeostatic model assessment-insulin resistance (n= 590)** | | | | | | |
| --- | --- | --- | --- | --- | --- | --- |
|  | **Q1 (n=118)** | **Q2 (n=118)** | **Q3(n=118)** | **Q4(n=118)** | **Q5 (n=118)** | **P-value**^†^ |
| **Characteristics** |  |  |  |  |  |  |
| **Age at blood draw (y)** | 41.0 (32.3-51.0) | 39.0 (28.0-52.0) | 39.5 (30.0-51.0) | 45.0 (32.3-55.0) | 47.5 (38.0-55.0) | 0.006 |
| **Sex** |  |  |  |  |  |  |
| Male | 73 (62) | 68 (58) | 71 (60) | 65 (55) | 50 (42) | 0.02 |
| **BMI (kg/m^2^)** | 24.2 (22.6-26.7) | 23.9 (22.3-26.6) | 24.7 (23.3-26.7) | 26.5 (24.6-29.0) | 30.0 (26.8-33.3) | < 0.001 |
| **Waist circumference (cm)** |  |  |  |  |  |  |
| Male | 85.0 (81.0-89.3) | 84.7 (82.0-91.0) | 85.0 (82.4-91.3) | 92.7 (86.0-100.6) | 99.4 (90.0-105.5) | < 0.001 |
| Female | 77.0 (72.0-83.0) | 76.6 (72.0-82.6) | 80.0 (73.0-85.0) | 83.0 (74.2-88.5) | 94.7 (86.2-101.0) | < 0.001 |
| **Fasting glucose (mg/dl)** | 79.0 (71.3-83.0) | 82.0 (78.0-85.0) | 86.0 (81.1-89.0) | 88.0 (84.0-92.0) | 92.5 (87.6-97.5) | < 0.001 |
| **Fasting insulin (mU/L)** | 5.3 (4.7-6.0) | 7.0 (6.5-7.4) | 8.0 (7.7-8.6) | 9.8 (9-10.4) | 14.0 (12.0-17.1) | < 0.001 |
| * Median (IQRs) or n (%) | | | | | | |

| Supplementary Table 5- Partial correlation**^*^** between cholesterol esters and lipids and glycemic measurements | | | | | | | |
| --- | --- | --- | --- | --- | --- | --- | --- |
| **Metabolite** | **HDL** | **LDL** | **TG** | **Total cholesterol** | **Insulin** | **Glucose** | **HOMA_IR** |
| CE (16:1) | **0.33** | **0.33** | 0.01 | **0.38** | **0.07** | 0.03 | **0.07** |
| CE (18:1) | **0.51** | **0.32** | **-0.60** | **0.22** | **-0.37** | **-0.20** | **-0.35** |
| CE (18:2) | **0.41** | **0.40** | **-0.53** | **0.29** | **-0.29** | **-0.15** | **-0.27** |
| CE (18:3) | **0.39** | **0.41** | **-0.27** | **0.39** | **-0.12** | **-0.03** | **-0.11** |
| CE (20:3) | **0.40** | **0.29** | **-0.54** | **0.15** | **-0.12** | **-0.15** | **-0.13** |
| CE (20:4) | **0.37** | **0.37** | **-0.42** | **0.29** | **-0.18** | **-0.05** | **-0.16** |
| CE (20:5) | **0.30** | **0.39** | **-0.10** | **0.42** | -0.03 | **0.06** | -0.01 |
| CE (22:6) | **0.25** | **0.31** | **-0.24** | **0.26** | **-0.13** | **-0.04** | **-0.12** |
| * Bolded correlation coefficient values indicate significant correlations (*p*<0.05). Correlations were adjusted for sex, age, body mass index and center.  Abbreviations: *CE=* cholesteryl ester, *HDL*= high density cholesterol, *LDL*= low density cholesterol, *TG*= triglyceride, *HOMA_IR*= Homeostatic Model Assessment of Insulin Resistance | | | | | | | |

| 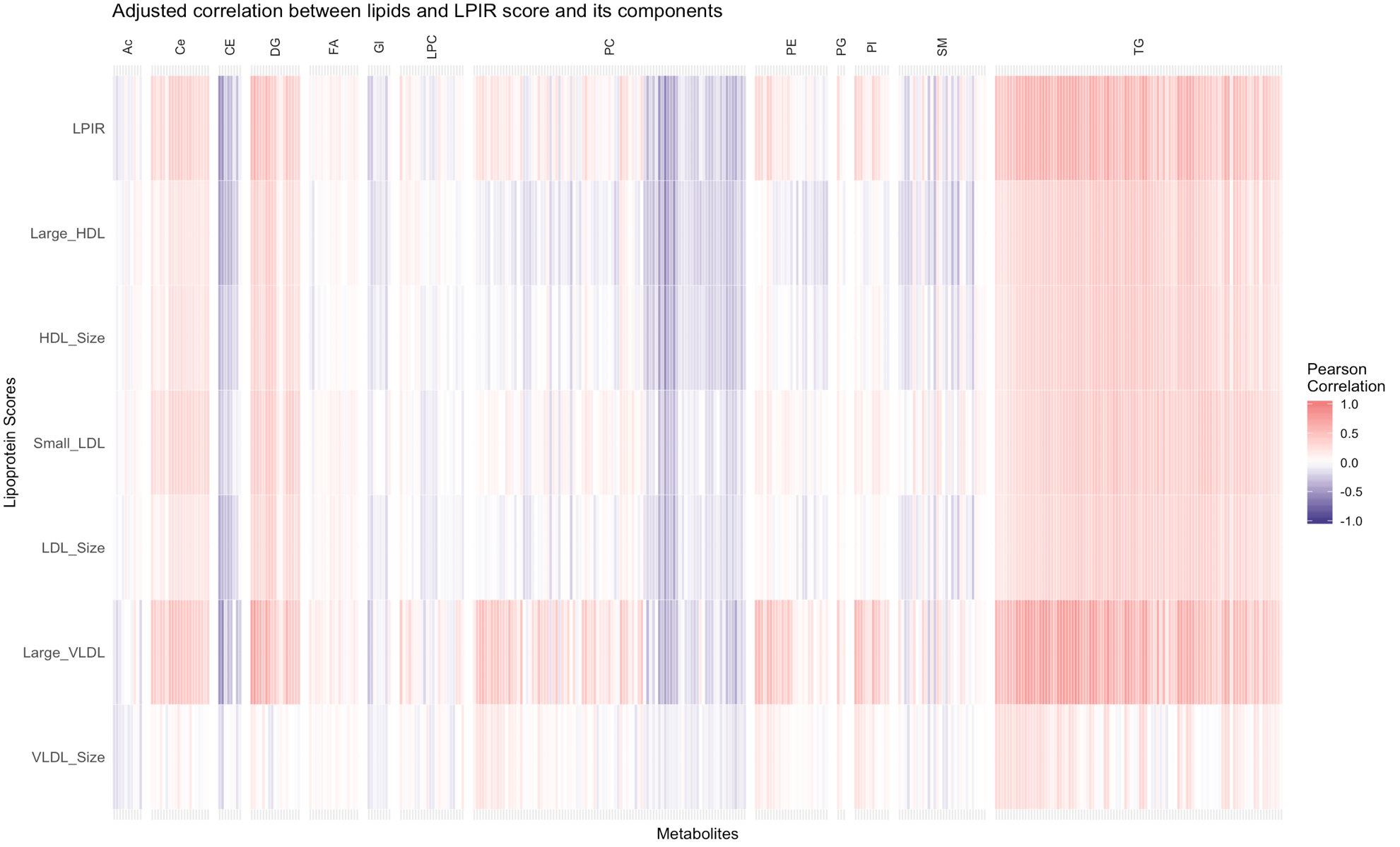 |
| --- |
| Supplementary Figure 1- Heat map showing positive (in red), and negative (in purple) partial correlations (adjusting for sex, age, BMI and center) of the lipoprotein insulin resistance (LPIR) score and its component scores with LPIR-correlated metabolites (n=363); metabolites were characterized according to their molecular structure. Each line belongs to one metabolite.  Abbreviations: *AC*= Acylcarnitine, *CE=* cholesteryl ester*, DG=* diglycerides*, FA=* Fatty acid*, LPC=* lysophosphatidylcholine*, LPE=* lysophosphoethanolamine*, PC=* phosphatidylcholine, *PE=* phosphoethanolamine*, PG=* prostaglandin, *PI=* phosphatidylinositol*, PS=* phosphatidylserine*, SM=* sphingomyelin*, TG=* triglycerides*.* |

| 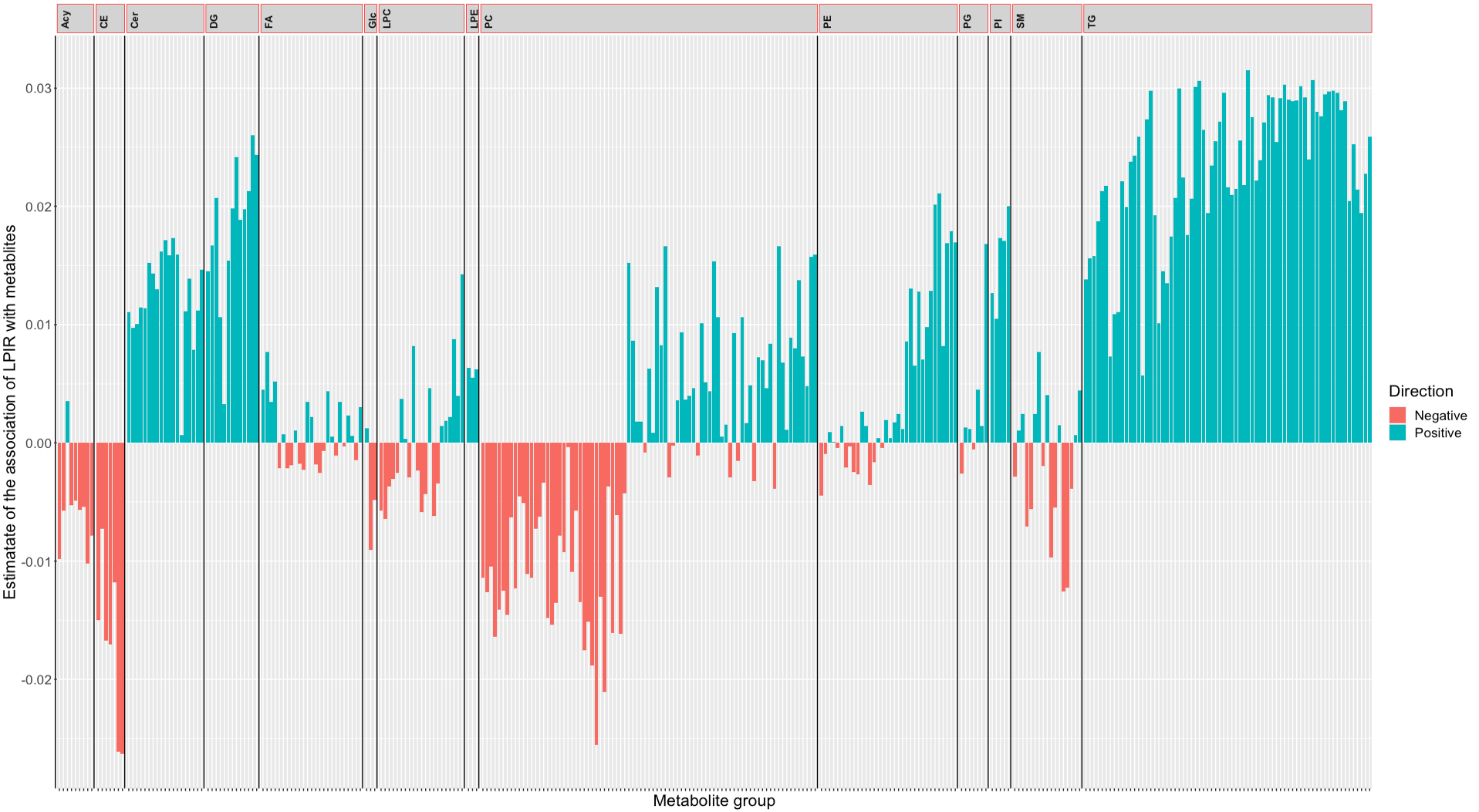 |
| --- |
| Supplementary Figure 2- Bar plot showing positive (in blue) and negative (in red) effect size derived from linear mixed models of the significant lipoprotein insulin resistance (LPIR)-related metabolites (n=319) characterized with respect to metabolite composition. Each line belongs to one metabolite. To have a better visualization groups with one metabolite (cholesterol and lactosylceramide (d18:1/24:1(15Z))) were not included in the figure.  Abbreviations: *Acy*= Acylcarnitine, *CE=* cholesteryl ester*, Cer=* Ceramide, *DG=* diglycerides*, FA=* Fatty acid*, Glc=* Glucosylceramide, *LPC=* lysophosphatidylcholine*, LPE=* lysophosphoethanolamine*, PC=* phosphatidylcholine, *PE=* phosphoethanolamine*, PG=* prostaglandin, *PI=* phosphatidylinositol*, PS=* phosphatidylserine*, SM=* sphingomyelin*, TG=* triglycerides*.* |
